# METTL7A‐Dependent m^6^A Methylation Orchestrates CXCR4^hi^ Neutrophil Pathogenicity and NETosis in Skin Inflammation

**DOI:** 10.1002/mco2.70941

**Published:** 2026-08-17

**Authors:** Wanting Liu, Jinkai Liang, Huiyi Quan, Xuan Liu, Kang Li, Xin Tang, Jiaqi Wang, Yunfeng Gu, Yaxing Bai, Ke Xue, Zhiguo Li, Xiaolei Su, Erle Dang, Jiaoling Chen, Gang Wang, Shuai Shao

**Affiliations:** ^1^ Department of Dermatology, Xijing Hospital Fourth Military Medical University Xi'an China

**Keywords:** METTL7A, *N* ^6^‐methyladenosine, neutrophil extracellular traps, psoriasis, phospholipid scramblase 1

## Abstract

Neutrophil extracellular traps (NETs) released by C–X–C motif chemokine receptor 4‐high (CXCR4^hi^) neutrophils are major contributors to skin inflammation in psoriasis, yet the upstream epitranscriptomic mechanisms that regulate NETosis remain poorly defined. Here, we investigated the role of methyltransferase‐like protein 7A (METTL7A) in *N* ^6^‐methyladenosine (m^6^A)‐mediated neutrophil activation and psoriasis pathogenesis. Using transcriptomic profiling of CXCR4^hi^ neutrophils from psoriasis patients, m^6^A RNA immunoprecipitation, in vitro functional assays, and in vivo murine models of skin inflammation, along with genetic silencing and adoptive transfer experiments, we demonstrated that METTL7A was preferentially upregulated in pathogenic and aged CXCR4^hi^ neutrophils and was positively associated with disease severity. METTL7A expression also increased during human promyelocytic leukemia cells(HL‐60 cell differentiation, maturation, and activation. Silencing *METTL7A* inhibited neutrophil maturation and aging, reduced NETosis, and alleviated psoriasiform inflammation in vivo. Adoptive transfer of *METTL7A*‐deficient neutrophils further protected mice from skin inflammation. Mechanistically, METTL7A stabilized phospholipid scramblase 1 (*PLSCR1*) mRNA through m^6^A‐associated regulation, thereby enhancing reactive oxygen species production and neutrophil extracellular traps (NETs) formation. These findings identify METTL7A as a novel epitranscriptomic regulator of pathogenic CXCR4^hi^ neutrophils, acting through the METTL7A–PLSCR1 axis to drive NETosis and skin inflammation, thereby providing a rationale for developing m^6^A‐targeted therapies in neutrophil‐associated disorders.

## Graphical abstract

In psoriasis, METTL7A is upregulated in aged CXCR4^hi^ neutrophils, where it stabilizes *PLSCR1* mRNA via m^6^A modification to enhance reactive oxygen species (ROS) production and neutrophil extracellular trap (NET) formation, thereby exacerbating disease severity. Genetic deletion or silencing of *METTL7A* suppresses neutrophil activation and NETosis, alleviating psoriasiform inflammation and underscoring the METTL7A–PLSCR1 axis as a potential anti‐inflammatory therapeutic target.

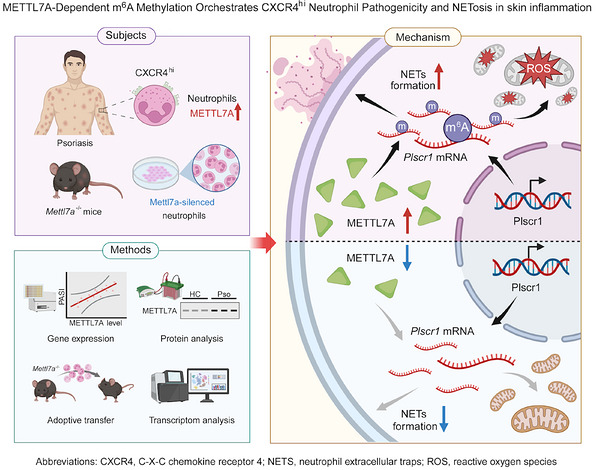

## 1 Introduction

Psoriasis is a chronic inflammatory skin disease characterized by epidermal hyperplasia, aberrant keratinocyte activation, and persistent immune‐cell infiltration. Although therapies targeting the interleukin‐23/interleukin‐17 axis have substantially improved disease control, the mechanisms that initiate and sustain local inflammatory amplification remain incompletely understood. Neutrophils are among the earliest and most abundant leukocytes recruited to psoriatic lesions and are increasingly recognized as active inflammatory effectors rather than passive bystanders [1, 2]. Activated neutrophils can undergo NETosis and release neutrophil extracellular traps (NETs), which are extracellular chromatin structures decorated with granular proteins [3]. In psoriasis, excessive NET formation can amplify keratinocyte activation through IL‐36‐ and Toll‐like receptor 4‐dependent signaling, thereby promoting a self‐sustaining inflammatory circuit [4]. Our group recently identified a disease‐relevant aged neutrophil subset characterized by C‐X‐C motif chemokine receptor 4‐high (CXCR4^hi^) expression, enrichment in the blood and skin of patients with psoriasis, correlation with disease severity, and heightened effector functions [5]. However, the upstream mechanisms that regulate aged CXCR4^hi^ neutrophil pathogenicity remain unclear.

*N* ^6^‐methyladenosine (m^6^A), the most abundant internal modification of mammalian mRNA, regulates RNA fate and has emerged as an important layer of immune‐cell control [6, 7, 8]. Classical m^6^A‐modifying enzymes such as *METTL3* and *FTO* have been linked to lymphocyte [9, 10] and macrophage biology [11, 12], as well as neutrophil development, emergency granulopoiesis, trafficking, and inflammatory responses [13, 14]. For instance, m^6^A methylation of the *CSF3R* mRNA, regulated by the demethylase ALKBH5 in neutrophils, critically influences emergency granulopoiesis and neutrophil mobilization during infection [14]. Nevertheless, how m^6^A‐dependent programs regulate neutrophil heterogeneity and effector functions in chronic inflammatory skin disease remains poorly defined.

Compared with canonical m^6^A regulators, the functions of nonclassical m^6^A‐associated methyltransferases in neutrophils have received limited attention. Methyltransferase‐like protein 7A (METTL7A) has been linked to m^6^A‐dependent regulation of mesenchymal stem‐cell differentiation [15, 16], tissue homeostasis in the human cerebellum [17], and immune‐related pathways in pan‐cancer analyses [18], However, whether METTL7A participates in neutrophil activation, aging‐associated phenotypic remodeling, or NET formation has not been established. Given the enrichment of pathogenic CXCR4^hi^ neutrophils in psoriasis, we hypothesized that METTL7A may represent an upstream epitranscriptomic regulator connecting neutrophil aging‐associated states with inflammatory effector programs.

In this study, we performed transcriptomic profiling of CXCR4^hi^ neutrophils from patients with psoriasis and healthy controls (HC). Our data revealed a marked upregulation of the nonclassical m^6^A methyltransferase METTL7A in psoriatic CXCR4^hi^ neutrophils, along with enrichment of m^6^A‐modified transcripts. Functional studies demonstrated that METTL7A promotes neutrophil activation, NET formation, and reactive oxygen species (ROS) production, whereas *METTL7A* silencing or *Mettl7a* deficiency mitigates neutrophil aging, reduces their accumulation in inflamed skin, and alleviates psoriasiform skin inflammation. Mechanistically, *METTL7A* supported phospholipid scramblase 1 (*PLSCR1)* mRNA stability through m^6^A‐associated regulation, and *PLSCR1* contributed to METTL7A‐dependent ROS production and NET formation. These findings identify a previously unrecognized METTL7A–PLSCR1 epitranscriptomic axis that promotes pathogenic neutrophil programs in psoriasis and suggest a potential strategy for targeting NETotic inflammation in neutrophil‐driven skin disease.

## 2 Results

### 2.1 *METTL7A* Is Upregulated in Aged CXCR4^hi^ Neutrophils in Psoriasis

**FIGURE 1:**
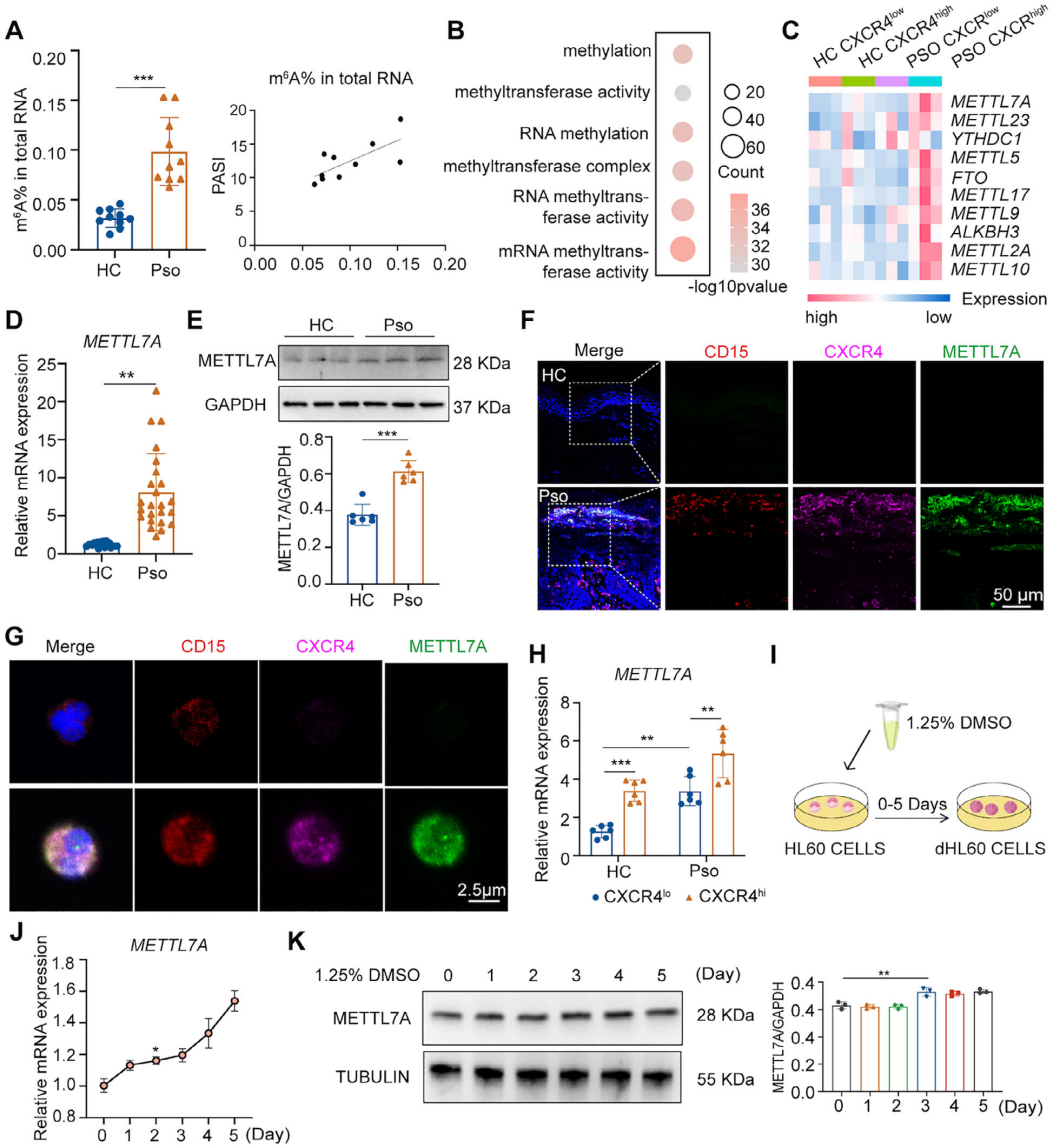
METTL7A‐mediated m^6^A RNA methylation is elevated in psoriatic neutrophils. (A) Left: Global m^6^A modification levels in total neutrophil RNA from healthy controls (HC) and patients with psoriasis (Pso) (*n* = 10). Right: Correlation between global m^6^A levels and Psoriasis Area and Severity Index (PASI) scores in patients with psoriasis. (B) Gene Ontology (GO) enrichment analysis of differentially expressed genes (DEGs) identified in CXCR4^hi^ and CXCR4^lo^ neutrophils isolated from the peripheral blood of healthy controls (HC) and patients with psoriasis (Pso). (C) Heatmap showing the expression of genes encoding RNA methyltransferases in CXCR4^hi^ and CXCR4^lo^ neutrophils from healthy controls (HC) and patients with Pso (Pso). (D) Relative *METTL7A* mRNA expression in peripheral blood neutrophils from healthy controls (HC) (*n* = 17) and patients with psoriasis (Pso) (*n* = 24). (E) Representative Western blot images and quantification of METTL7A protein expression in neutrophils from healthy controls (HC) and patients with psoriasis (Pso). GAPDH was used as a loading control. (F and G) Representative immunofluorescence staining of skin sections (F) and peripheral blood neutrophils (G) from healthy controls (HC) and patients with psoriasis (Pso) samples. METTL7A is shown in green, CXCR4 in magenta, CD15 in red, and nuclei were counterstained with DAPI (blue). Scale bars, 50 µm in F and 2.5 µm in G. (H) Relative *METTL7A* mRNA expression in CXCR4^hi^ and CXCR4^lo^ neutrophils isolated from healthy controls and patients with psoriasis (*n* = 6). (I) Schematic illustration of the in vitro differentiation model, in which HL‐60 cells were treated with 1.25% DMSO for 0–5 days to generate HL‐60‐derived neutrophil‐like cells. (J) Time‐course analysis of relative *METTL7A* mRNA expression during DMSO‐induced differentiation of HL‐60 cells differentiation from Day 0 to Day 5. (K) Representative Western blot images and quantification of METTL7A protein expression during DMSO‐induced HL‐60 cells differentiation from Day 0 to Day 5. GAPDH served as the loading control. Data are presented as mean ± SEM. Statistical significance was determined using unpaired Student's *t*‐test or two‐way ANOVA, as appropriate. ^**^ *p* < 0.01, ^***^ *p* < 0.001.

To determine whether m^6^A methylation contributes to neutrophil dysregulation in psoriasis, we first measured global m^6^A levels in neutrophil RNA using a colorimetric assay. Neutrophils from psoriasis patients exhibited significantly elevated m^6^A abundance compared with HC (*n* = 10), and these levels showed a positive correlation with Psoriasis Area and Severity Index (PASI) scores, indicating clinical relevance (Figure 1A). Reanalysis of our previously published neutrophil transcriptome dataset (HRA005230) further revealed enrichment of the m^6^A methylation pathway specifically in CXCR4^hi^ neutrophils (Figure 1B). Among all m^6^A‐related enzymes including *METTL10* and *FTO*, *METTL7A* emerged as the most significantly upregulated gene, as confirmed by differential gene expression heatmaps (Figure 1C) and volcano plots (Figure S1A). qRT‐PCR results confirmed that *METTL7A* was the most significantly upregulated gene among all m^6^A‐related enzymes (Figure 1D); in contrast, genes such as *METTL4* and *WTAP* exhibited only modest upregulation (Figure S1B). Consistently, Western blotting (Figure 1E) demonstrated overexpression of *METTL7A* in circulating neutrophils from psoriasis patients. Importantly, immunofluorescence analysis of lesional skin revealed strong METTL7A signals in CD15^+^CXCR4^hi^ neutrophils (Figures 1F and S1 C). To further investigate this, we performed colocalization analysis of METTL7A and the NETosis marker citrullinated histone H3 (CitH3) in psoriatic lesional tissues. The results showed that in METTL7A‐high neutrophils, CitH3 signals were also positive, and the two markers exhibited obvious colocalization (Figure S1D). Meanwhile, stratification of neutrophils based on CXCR4 expression levels demonstrated that both METTL7A immunofluorescence intensity (Figure 1G) and mRNA (Figure 1H) levels were significantly higher in CXCR4^hi^ neutrophils compared with CXCR4^lo^ subsets across both healthy and psoriatic cohorts, with the highest METTL7A expression observed in the psoriatic CXCR4^hi^ population (Figure S1E). Furthermore, in peripheral blood neutrophils collected from 10 psoriasis patients before and after biologic therapy, both mRNA and protein levels of METTL7A were significantly decreased after treatment (Figure S2A,B), and m^6^A modification levels were also markedly reduced (Figure S2C).

To assess whether *METTL7A* expression is associated with neutrophil differentiation, we induced HL‐60 cells to differentiate into neutrophil‐like cells (dHL‐60) with DMSO (Figure 1I). qRT‐PCR (Figure 1J) and Western blotting (Figure 1K) showed a time‐dependent increase in *METTL7A* expression, with transcript levels rising progressively during differentiation. Meanwhile, reanalysis of a published RNA‐seq dataset of human peripheral blood neutrophils (HRA005230) revealed that METTL7A mRNA expression levels were significantly higher in the CXCR4^hi^ neutrophil subset than in the CXCR4^lo^ subset, a pattern consistent with the expression trend of CD101 in these two subsets (Figure S2D). Moreover, upon stimulating neutrophils with serum from HC or psoriasis patients, we observed that psoriatic serum significantly upregulated METTL7A at both the mRNA and protein levels (Figure S2E,F). Together, these results establish that METTL7A‐dependent m^6^A methylation is a distinguishing feature of aged CXCR4^hi^ neutrophils, linking it to both neutrophil maturation and psoriasis progression.

### 2.2 Genetic Deletion of *Mettl7a* Confers Protection in an Imiquimod‐Induced Mouse Model

**FIGURE 2:**
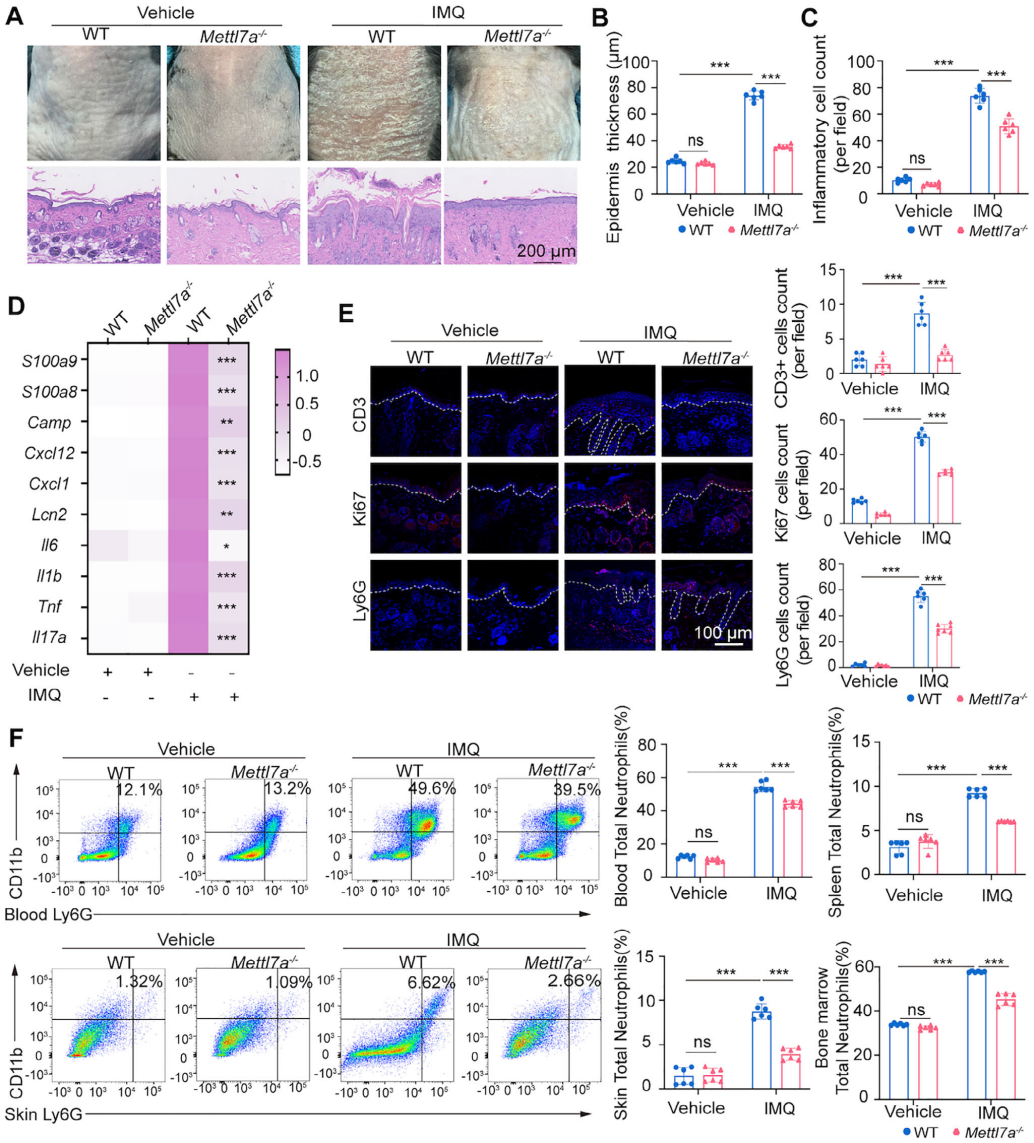
METTL7A deficiency attenuates IMQ‐induced psoriasiform skin inflammation. (A) Representative gross images (upper panels) and hematoxylin and eosin (H&E) staining (lower panels) of dorsal skin from wild‐type (WT) and *Mettl7a^−/−^* mice treated with vehicle or imiquimod (IMQ). Scale bar, 200 µm. (B) Quantification of epidermal thickness in dorsal skin from the indicated groups (*n* = 6). (C) Quantification of infiltrating inflammatory cells in dorsal skin from the indicated groups (*n* = 6). (D) Heatmap showing the relative mRNA expression of psoriasis‐associated inflammatory genes, including *S100a8*, *S100a9*, *Camp*, *Cxcl12*, *Cxcl1*, *Lcn2*, *Il6*, *Tnf*, and *Il17a*, in dorsal skin from the indicated groups, as determined by qRT‐PCR. Color intensity indicates relative expression levels (purple, high; white, low) (*n* = 6). (E) Representative immunofluorescence images and quantification of Ki67^+^ epidermal cells, Ly6G^+^ neutrophils, and CD3^+^ T cells in mouse skin. Scale bar, 100 µm (*n* = 6). (F) Representative flow cytometry plots of neutrophils in blood (upper panels) and skin (lower panels) from WT and *Mettl7a^−/−^* mice treated with vehicle or IMQ. Quantification of neutrophil frequencies in blood, spleen, skin, and bone marrow from the indicated groups is shown on the right (*n* = 6). Data are presented as mean ± SEM. Statistical significance was determined by two‐way ANOVA. ^*^ *p *< 0.05, ^**^ *p* < 0.01, ^***^ *p* < 0.001, and *ns*, not significant.

To determine the in vivo relevance of METTL7A, we generated *Mettl7a* knockout (*Mettl7a^−/−^*) mice using CRISPR/Cas9 technology (Figure S3A). Upon daily topical application of imiquimod (IMQ) for 5 days, WT mice developed pronounced erythema, scaling, and dermal thickening; in contrast, these murine skin inflammatory features were markedly attenuated in *Mettl7a^−/−^* littermates (Figure 2A). Histological analysis confirmed thinner acanthotic epidermis, fewer Munro microabscesses (Figure 2B), and reduced dermal leukocyte infiltration in *Mettl7a^−/−^* mice compared with WT controls (Figure 2C). At the molecular level, qRT‐PCR analysis of lesional skin revealed significant reductions in proinflammatory cytokines (*Il17a, Il1b, Il6, Tnf*), chemokine (*Lcn2, Cxcl1, Cxcl12)*, and antimicrobial peptide (*Camp, S100a8, S100a9*) in *Mettl7a^−/−^* mice (Figure 2D). Immunofluorescence staining revealed decreased keratinocyte proliferation (Ki67^+^ cells), along with reduced infiltration of CD3^+^ T cells and Ly6G^+^ neutrophils (Figure 2E). Flow cytometry further demonstrated a broad reduction in neutrophil frequencies across skin, blood (Figure 2F), bone marrow (Figure S4A), and spleen compartments in the absence of *Mettl7a* (Figure S4B). Collectively, these data indicate that genetic loss of *Mettl7a* protects against IMQ‐induced psoriasiform inflammation by limiting neutrophil activation, recruitment, and inflammatory mediator release.

### 2.3 Adoptive Transfer of *Mettl7a*‐Deficient Neutrophils Attenuates Murine Skin Inflammation

**FIGURE 3:**
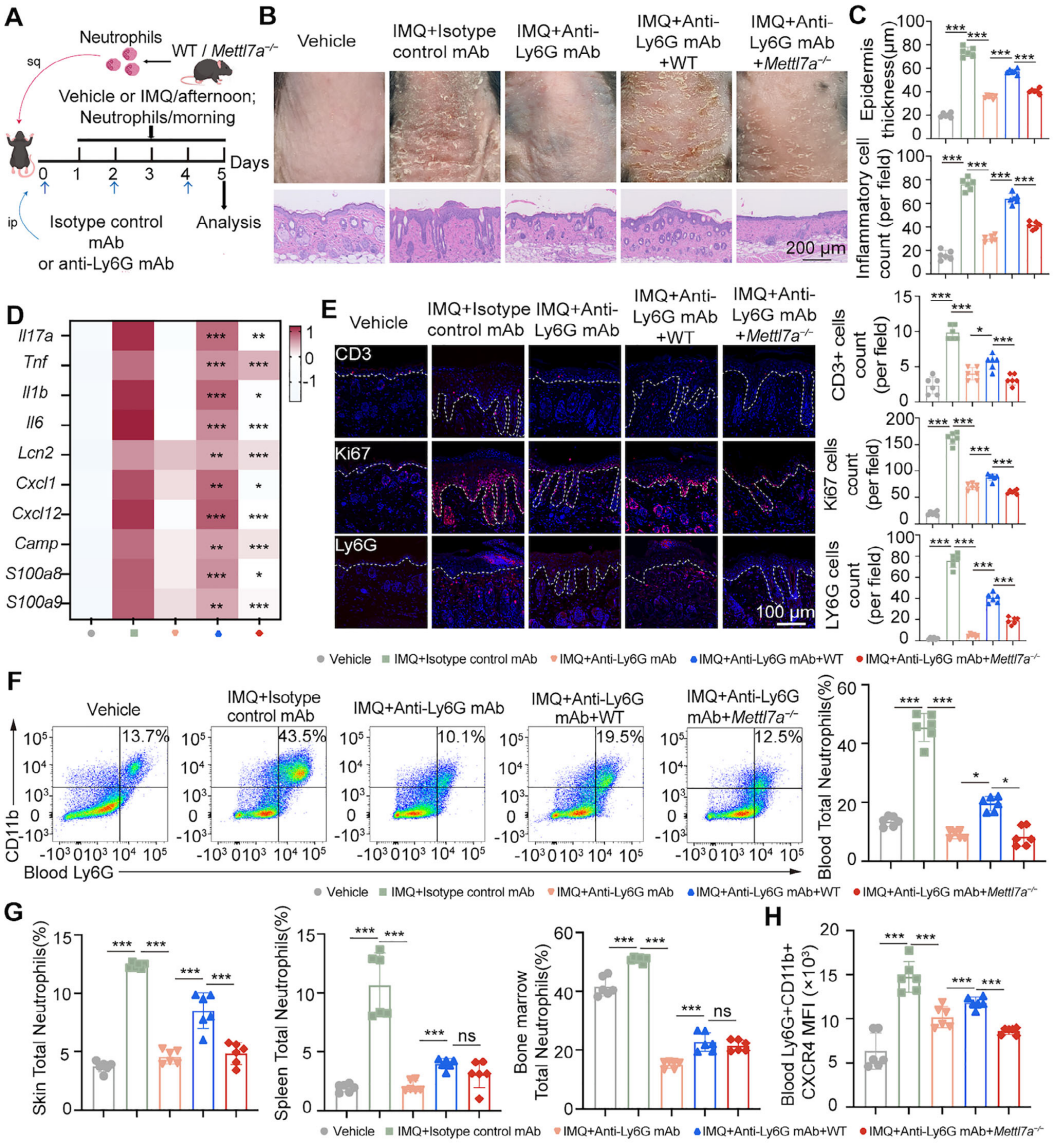
Adoptive transfer of *Mettl7a*‐deficient neutrophils attenuates IMQ‐induced skin inflammation. (A) Schematic illustration of the adoptive transfer protocol for wild‐type (WT) or *Mettl7a^−/−^* neutrophils in the IMQ‐induced skin inflammation model. (B) Representative gross images (upper panels) and H&E staining (lower panels) of dorsal skin from mice in the indicated treatment groups. Scale bar, 200 µm. (C) Quantification of epidermal thickness and infiltrating inflammatory cells in dorsal skin from the indicated groups (*n* = 6). (D) Heatmap showing the relative mRNA expression of *S100a8*, *S100a9*, Camp, *Cxcl12*, *Cxcl1*, *Lcn2*, *Il6*, *Tnf*, *Il1b*, and *Il17a*, in dorsal skin from the indicated groups, as determined by qRT‐PCR. Color intensity indicates relative expression levels (red, high; white, low) (*n* = 6). (E) Representative immunofluorescence images and quantification of CD3^+^ T cells, Ki67^+^ epidermal cells, and Ly6G^+^ neutrophils in mouse skin sections from the indicated groups (*n* = 6). Scale bar, 100 µm. (F) Representative flow cytometry plots of blood neutrophils and quantification of neutrophil frequencies in blood from the indicated groups (*n* = 6). (G) Quantification of neutrophil frequencies in skin, spleen, and bone marrow from the indicated groups (*n* = 6). (H) Mean fluorescence intensity (MFI) of CXCR4 on peripheral blood neutrophils from the indicated groups (*n* = 6). Data are presented as mean ± SEM. Statistical significance was determined by one‐way ANOVA followed by appropriate posthoc tests. ^*^ *p *< 0.05, ^**^ *p* < 0.01, ^***^ *p* < 0.001, and *ns*, not significant.

To directly assess the contribution of neutrophil‐intrinsic Mettl7a to skin inflammation, we performed neutrophil adoptive transfer experiments (Figure 3A). Mice were first depleted of endogenous neutrophils using anti‐Ly6G antibodies, then reconstituted with Ly6G^+^ neutrophils isolated from WT or *Mettl7a^−/−^* donors (Figure 3B). Histological analysis confirmed that the transfer of WT neutrophils significantly exacerbated IMQ‐induced skin inflammation, whereas recipients of *Mettl7a^−/−^* neutrophils exhibited markedly reduced epidermal hyperplasia and dermal leukocyte infiltration (Figure 3C). qRT‐PCR analysis showed that lesional skin from *Mettl7a^−/−^* neutrophil recipients expressed significantly lower levels of proinflammatory cytokines (*Il17a, Il1b, Il6, Tnf*), chemokine (*Lcn2, Cxcl1, Cxcl12)*, and antimicrobial peptide (*Camp*, *S100a8*, *S100a9*) compared with those receiving WT donor neutrophils (Figure 3D). Immunofluorescence staining further revealed decreased keratinocyte proliferation (Ki67^+^ cells), along with reduced infiltration of CD3^+^ T cells and Ly6G^+^ neutrophils in recipients of *Mettl7a^−/−^* neutrophils (Figure 3E). Flow cytometric analysis confirmed that endogenous neutrophils were effectively depleted after anti‐Ly6G mAb injection in peripheral blood (Figures 3F and S5A), skin, and target tissues (Figure 3G). Following the transfer of WT donor neutrophils, the repopulated neutrophils demonstrated normal migration and circulation‐associated aging capabilities as defined by multimarker surface phenotype (Figure S5B). In contrast, transfer of *Mettl7a^−/−^* neutrophils resulted in impaired migration and aging, as evidenced by reduced neutrophil numbers in peripheral blood and decreased CXCR4 expression levels (Figures 3H and S5C).

### 2.4 METTL7A Promotes Neutrophil Aging, Effector Function, and NET Formation

**FIGURE 4:**
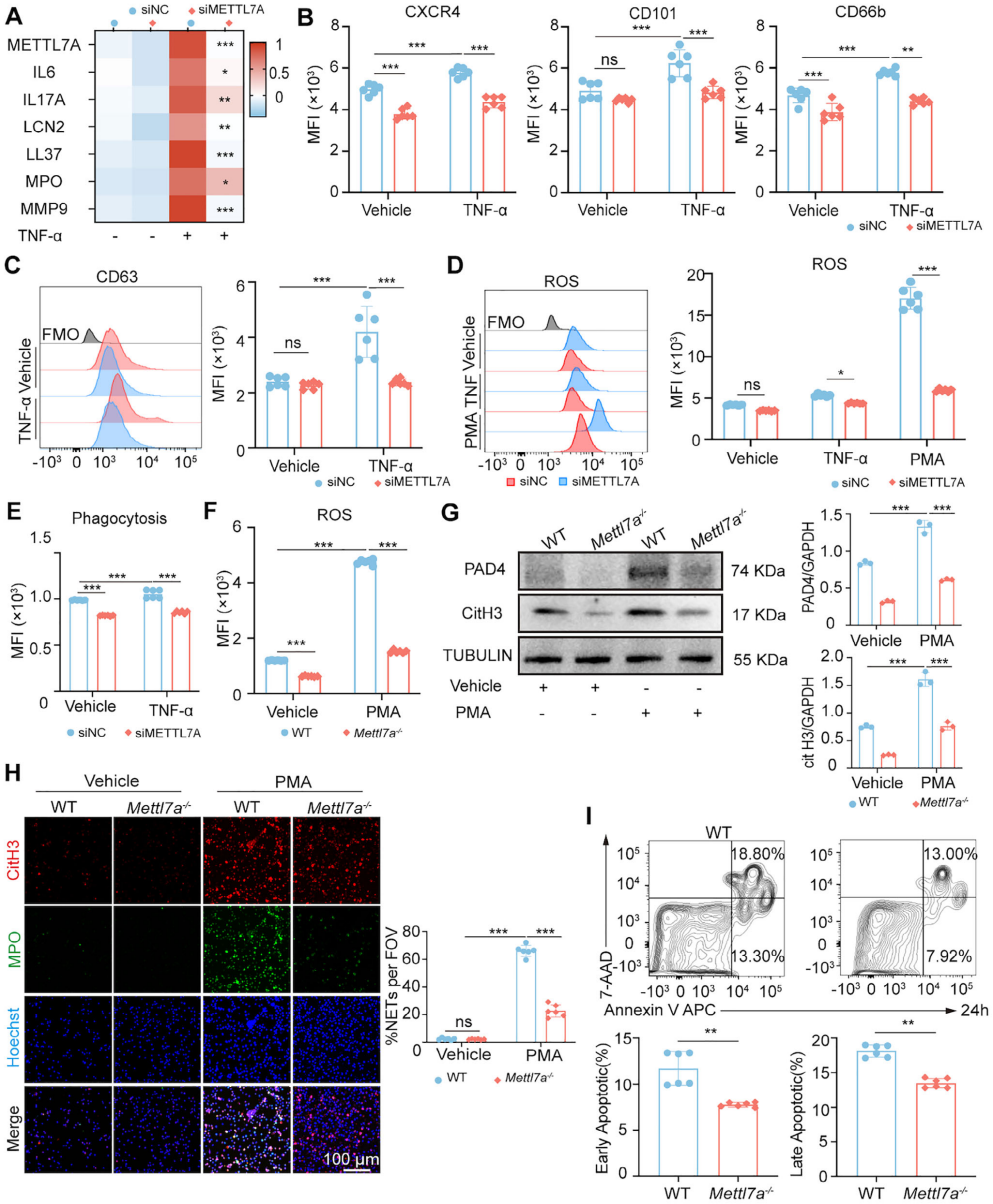
METTL7A regulates neutrophil‐like maturation, inflammatory effector functions, and aging‐associated marker dynamics. (A) Heatmap showing the relative mRNA expression of *METTL7A*, *IL6*, *IL17A*, *LCN2*, *LL37*, *MPO*, and *MMP9* in dHL‐60 neutrophil‐like cells transfected with control siRNA (siNC) or *METTL7A* siRNA (siMETTL7A), with or without TNF‐α stimulation, as determined by qRT‐PCR. Color intensity indicates relative expression levels (red, high; blue, low) (*n* = 6). (B) Flow cytometric analysis and quantification of CXCR4, CD101, and CD66b expression in dHL‐60 neutrophil‐like cells under the indicated conditions (*n* = 6). (C) Flow cytometric analysis and quantification of neutrophil degranulation, as assessed by CD63 expression, in dHL‐60 neutrophil‐like cells under the indicated conditions (*n* = 6). (D) Measurement of reactive oxygen species (ROS) production in dHL‐60 neutrophil‐like cells following TNF‐α or PMA stimulation under the indicated conditions (*n* = 6). (E) Flow cytometric analysis of phagocytosis of pHrodo Green E. coli particles by dHL‐60 neutrophil‐like cells under the indicated conditions (*n* = 6). (F) Measurement of ROS production in wild‐type (WT) and *Mettl7a^−/−^* neutrophils under vehicle or PMA (phorbol 12‐myristate 13‐acetate) stimulation (*n* = 6). (G) Representative Western blot images and quantification of PAD4 and citrullinated histone H3 (CitH3) in wild‐type (WT) and *Mettl7a^−/−^* neutrophils treated with vehicle or PMA. TUBULIN served as the loading control. (H) Representative nonpermeabilized immunofluorescence images showing extracellular MPO (green) /CitH3 (red)‐associated NET‐like structures and Hoechst (blue)‐stained DNA, together with morphology‐based quantification of NET‐positive cells in wild‐type (WT) and *Mettl7a^−/−^* neutrophils (*n* = 6). Scale bar, 25 µm. (I) Representative Annexin V/7‐AAD flow cytometry plots and quantification of early and late apoptotic neutrophils from wild‐type (WT) and *Mettl7a^−/−^* mice (*n* = 6). Data are presented as mean ± SEM. Statistical significance was determined by two‐way ANOVA, as appropriate. ^*^ *p *< 0.05, ^**^ *p* < 0.01, ^***^ *p* < 0.001, and *ns*, not significant.

To investigate how METTL7A regulates neutrophil function, we silenced *METTL7A* in dHL‐60 cells with/without TNF‐α stimulation. Silencing efficiency was confirmed by qRT‐PCR and immunoblotting (Figure S6A,B). TNF‐α stimulation induced robust upregulation of METTL7A (Figures S6C and S7A), accompanied by increased expression of proinflammatory cytokines (*IL17A, IL1B, IL6, TNF*), chemokine (*CXCL1)*, antimicrobial effector molecules (*MPO, LCN2, CAMP*), and migration molecules (*MMP9*) (Figure 4A). These inductions were significantly attenuated following *METTL7A* silencing. Flow cytometry analysis showed that *METTL7A* silencing reduced the expression of maturation markers in dHL‐60 neutrophil‐like cells (CXCR4, CD66b, CD101) (Figures 4B and S8A). Functional assays revealed diminished degranulation capacity, as indicated by reduced CD63 expression (Figure 4C). Since ROS are critical mediators of NETosis, we quantified intracellular ROS using the DCFH‐DA probe and found that ROS levels were significantly suppressed in *METTL7A*‐deficient dHL‐60 neutrophil‐like cells (Figures 4D and S8B). Additionally, *METTL7A* silencing also impaired phagocytic uptake of *E. coli* bioparticles (Figure 4E).

To validate the above findings in a more physiologically relevant system, we further analyzed primary bone marrow neutrophils isolated from WT and *Mettl7a^−/−^* mice (Figure S9A). Consistently, DCFH‐DA staining revealed markedly reduced ROS production in *Mettl7a^−/−^* neutrophils (Figures 4F and S9B). Moreover, *Mettl7a^−/−^* primary neutrophils exhibited significantly impaired NET formation, as indicated by reduced levels of PAD4 and extracellular CitH3 in Western blot analysis (Figure 4G), decreased signal intensity in nonpermeabilized MPO immunofluorescence, lower extracellular CitH3 levels (Figure 4H), and diminished SYTOX Green fluorescence (Figure S9C). After 24 and 48 h of in vitro culture, Annexin V/7‐AAD staining demonstrated that both early and late apoptotic rates were significantly lower in *Mettl7a^−/−^* neutrophils compared with WT controls (Figures 4I and S9D), indicating that METTL7A deficiency delays spontaneous neutrophil apoptosis. Furthermore, flow cytometry analysis of the dynamic changes in aging‐ and migration‐related surface markers showed that in WT neutrophils, CXCR4 and CD101 were upregulated over time, whereas CD62L was gradually downregulated. In contrast, *Mettl7a^−/−^* neutrophils exhibited impaired time‐dependent upregulation of CXCR4 and CD101, as well as a delayed downregulation of CD62L (Figure S9E). Collectively, these primary cell‐based results are highly consistent with the findings obtained from dHL‐60 neutrophil‐like cells and further support a critical role for METTL7A in regulating neutrophil aging, effector functions, and NETosis, processes that may represent key contributors to the pathogenic role of neutrophils in psoriasis.

### 2.5 METTL7A Orchestrates the Transcriptional Landscape of Neutrophil Activation

**FIGURE 5:**
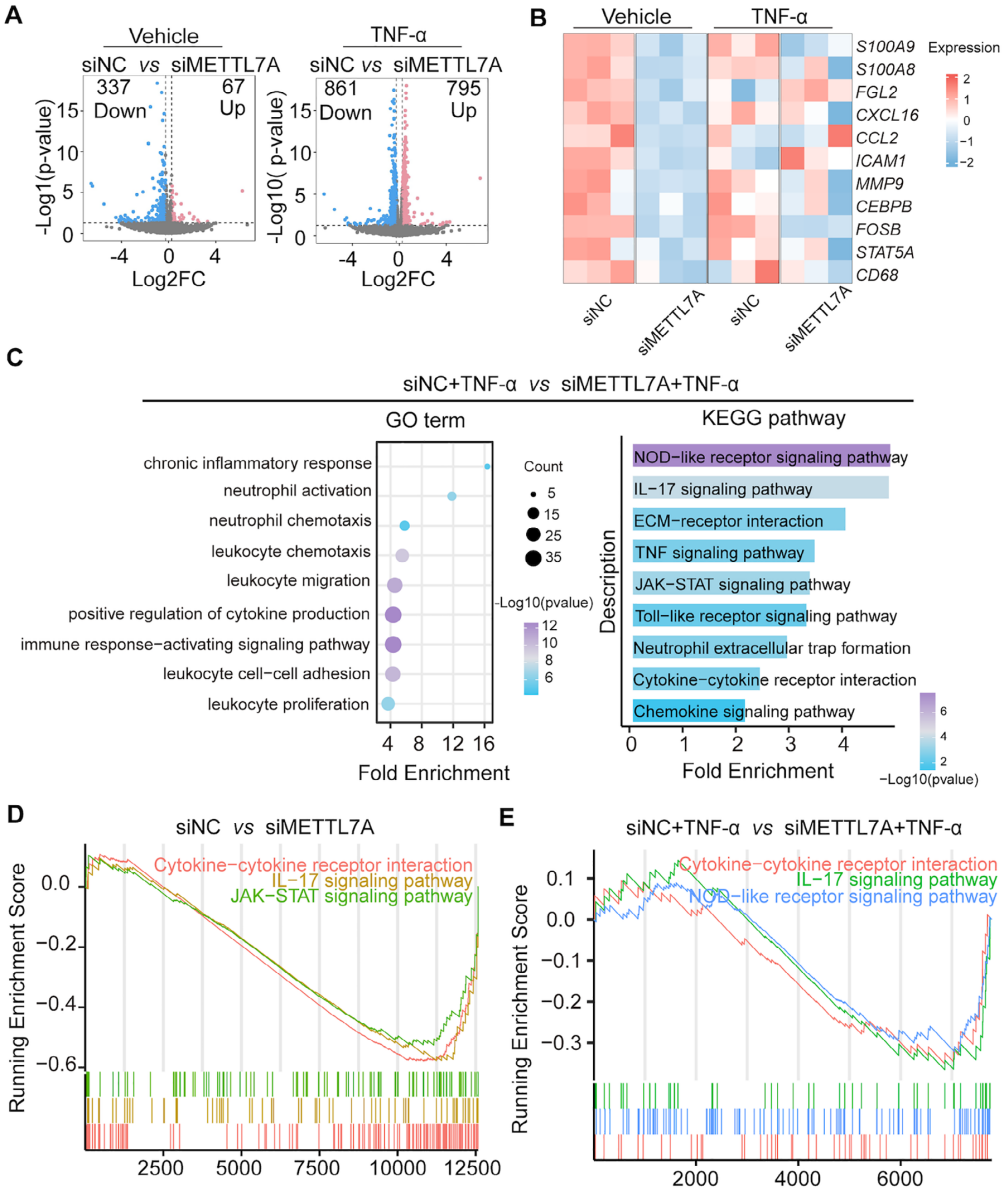
Transcriptomic profiling reveals that METTL7A regulates neutrophil inflammatory and signaling pathways. (A) Volcano plots of DEGs in dHL‐60 neutrophil‐like cells transfected with siNC or siMETTL7A under vehicle‐treated (left) or TNF‐α‐stimulated (right) conditions. (B) Heatmap of representative DEGs involved in dHL‐60 neutrophil‐like cells activation and inflammatory responses across the indicated groups. (C) GO and Kyoto Encyclopedia of Genes and Genomes (KEGG) pathway enrichment analyses of DEGs identified in TNF‐α‐stimulated dHL‐60 neutrophil‐like cells comparing siNC + TNF‐α and siMETTL7A + TNF‐α. (D and E) Gene Set Enrichment Analysis (GSEA) showing enrichment of inflammatory signaling pathways in dHL‐60 neutrophil‐like cells comparing siNC and siMETTL7A under basal conditions (D) and TNF‐α‐stimulated conditions (E).

To delineate the broader transcriptional programs controlled by METTL7A, we performed RNA sequencing of *METTL7A*‐silenced and control neutrophils (HRA013229). Volcano plots showed that in unstimulated cells, a total of 414 genes were differentially expressed (67 upregulated and 337 downregulated), while TNF‐α stimulation expanded this effect to 1656 genes (795 upregulated and 861 downregulated) (Figure 5A). Heatmap analysis demonstrated that key neutrophil effector genes such as *S100A9*, *S100A8*, *MMP9*, *PLSCR1*, *FOSB*, and *CEBPB* were significantly downregulated (Figure 5B). GO analysis indicated that genes suppressed under basal conditions were enriched in proteasomal activity, ribosome biogenesis, and oxidative phosphorylation pathways (Figure S10A). Under TNF‐α stimulation, *METTL7A* silencing suppressed pathways associated with NOD‐like receptor signaling, IL‐17 signaling, and extracellular matrix interactions. KEGG analysis further highlighted impairments in neutrophil‐mediated cytotoxicity, chemotaxis, and chemokine signaling (Figure 5C). Gene Set Enrichment Analysis (GSEA) confirmed broad repression of cytokine‐receptor interactions, IL‐17 signaling, and JAK–STAT pathways (Figure 5D,E). Taken together, these results identify METTL7A as a transcriptional hub that sustains neutrophil maturation, proinflammatory signaling, and CXCR4‐driven aging phenotypes.

### 2.6 The METTL7A–PLSCR1 Axis Drives Neutrophil NETosis Through M^6^A‐Dependent Regulation

**FIGURE 6:**
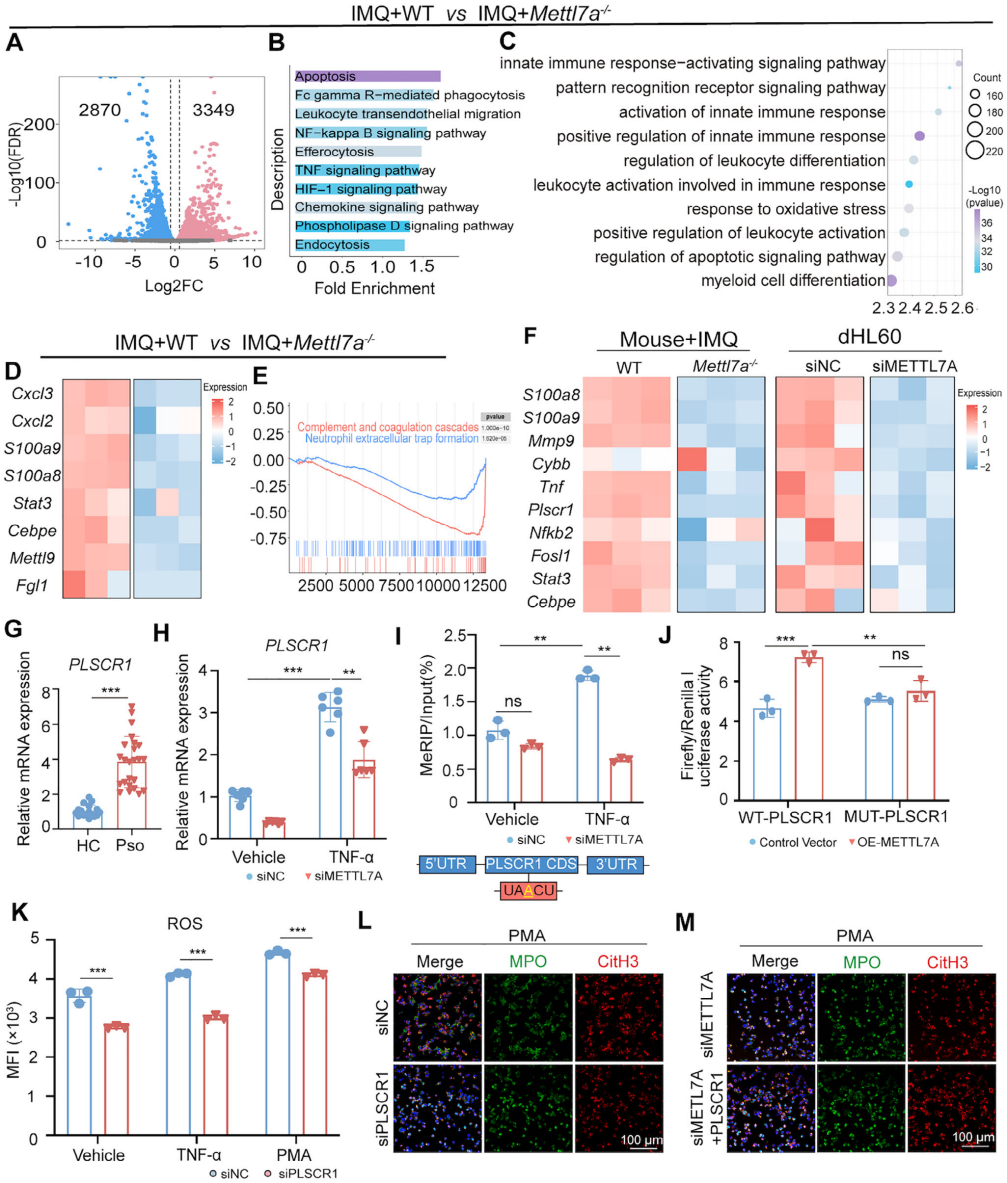
METTL7A deficiency impairs neutrophil inflammatory programs through regulation of PLSCR1. (A) Volcano plot showing DEGs in neutrophils isolated from IMQ‐treated wild‐type (WT) and *Mettl7a* ^−/−^ mice. (B) KEGG pathway enrichment analysis of DEGs identified in the comparison of IMQ‐treated WT and *Mettl7a* ^−/−^ neutrophils. (C) GO enrichment analysis of DEGs identified in the comparison of IMQ‐treated wild‐type (WT) and *Mettl7a* ^−/−^ neutrophils. (D) Heatmap of representative DEGs (*Cxcl3*, *Cxcl2*, *S100a9*, *Stat3*, *Mettl9*, *Fgf11*) in neutrophils from IMQ‐treated WT and *Mettl7a* ^−/−^ mice. (E) GSEA showing enrichment of selected pathways in neutrophils from IMQ‐treated WT and *Mettl7a* ^−/−^ mice. (F) Heatmaps of representative neutrophil‐related genes in two models: neutrophils from IMQ‐treated wild‐type (WT) and *Mettl7a* ^−/−^ mice (left) and dHL‐60 neutrophil‐like cells transfected with siNC or siMETTL7A (right). (G) Relative mRNA expression of *PLSCR1* in neutrophils from healthy controls (HC) (*n* = 17) and patients with psoriasis (Pso) (*n* = 24). (H) Relative mRNA expression of *PLSCR1* in dHL‐60 neutrophil‐like cells transfected with siNC or siMETTL7A, with or without TNF‐α stimulation (*n* = 6). (I) Methylated RNA immunoprecipitation followed by qPCR (MeRIP–qPCR) analysis of m^6^A modification on *PLSCR1* mRNA in dHL‐60 neutrophil‐like cells under the indicated conditions. (J) Dual‐luciferase reporter assay showing relative firefly/Renilla luciferase activity of wild‐type (WT) or mutant (MUT) PLSCR1 reporter constructs in the presence of control vector or METTL7A overexpression vector (OE‐METTL7A) (*n* = 3). (K) Measurement of ROS production in dHL‐60 neutrophil‐like cells transfected with siNC or siPLSCR1 under vehicle, TNF‐α, or PMA stimulation (*n* = 3). (L and M) Representative immunofluorescence images of MPO (green), CitH3 (red), and Hoechst‐stained nuclei (blue) in PMA‐stimulated dHL‐60 neutrophil‐like cells transfected with siNC or siPLSCR1 (L), or cotransfected with siMETTL7A and a PLSCR1 overexpression construct (M). Scale bars, 100 µm. Data are presented as mean ± SEM. Statistical significance was determined by unpaired Student's *t*‐test or two‐way ANOVA, as appropriate. ^**^ *p* < 0.01, ^***^ *p* < 0.001, and *ns*, not significant.

To identify downstream effectors of METTL7A, we integrated RNA‐seq data from murine *Mettl7a* ^−^ *^/–^* neutrophils (accession number CRA029900) and human METTL7A‐silenced dHL‐60 neutrophil‐like cells (accession number HRA013229). Volcano plot results showed that in the IMQ‐treated group, a total of 2870 genes were significantly downregulated in *Mettl7a* ^–/–^ neutrophils compared with WT controls (fold change > 1.5, FDR < 0.05) (Figure 6A). Gene ontology analysis indicated that *Mettl7a*‐deficient neutrophils exhibited altered metabolic and immune response pathways, with NET formation being one of the most significantly affected processes (Figure 6B,C). Heatmap visualization demonstrated that suppressed genes included those associated with neutrophil differentiation and activation (*Mmp9, Cebpb*), antibacterial (*S100a8, S100a9*), and chemotaxis (*Cxcl2*, *Cxcl3*) (Figure 6D). GSEA further validated this finding (Figure 6E). Through a multistep screening strategy (transcriptomic overlap with predicted m^6^A consensus‐containing transcripts, GO/KEGG functional annotation, and qRT‐PCR validation), cross‐species analysis revealed a set of conserved downregulated genes including *Cybb*, *Plscr1, Fosl1, Stat3*, and *Cebpe*, among which *Plscr1* emerged as a prominent candidate (Figure 6F), given its reported roles in cytokine production, ROS generation, and NETosis [19].

Consistently, qRT‐PCR (Figure 6G) and Western blotting (Figure S11A) confirmed that *PLSCR1* expression was elevated in neutrophils from psoriasis patients compared with HC. Moreover, after effective clinical treatment, both PLSCR1 mRNA and protein levels in patient neutrophils were markedly reduced (Figure S11B,C). Stimulation of dHL‐60 neutrophil‐like cells or primary human neutrophils with serum from psoriasis patients similarly induced a significant upregulation of *PLSCR1* mRNA and protein expression (Figure S11*D*,E), indicating that psoriasis‐associated inflammatory factors promote PLSCR1 expression. Furthermore, during HL‐60 cell differentiation, *PLSCR1* mRNA and protein levels gradually increased with differentiation time (Figure S11*F*,G), consistent with the expression pattern of METTL7A (Figure 1J,K). In *METTL7A*‐silenced dHL‐60 neutrophil‐like cells, both mRNA and protein levels of PLSCR1 were significantly reduced, as determined by qRT‐PCR and Western blot, respectively (Figures 6,H and S11H). MeRIP–qPCR demonstrated a significant reduction of m^6^A‐modified *PLSCR1* transcripts in *METTL7A*‐silenced neutrophils, supporting PLSCR1 as a downstream epitranscriptomic target of METTL7A (Figures 6I and S12A); this was further validated by luciferase reporter assays showing that METTL7A overexpression enhanced the activity of the WT‐PLSCR1 but not a mutant lacking the predicted m^6^A site (Figure 6J). Moreover, METTL7A deficiency significantly shortened the half‐life of PLSCR1 mRNA (Figure S13A), indicating that METTL7A‐mediated m^6^A modification enhances PLSCR1 transcript stability. Following confirmation of *PLSCR1* silencing efficiency by qRT‐PCR and immunoblotting (Figure S13B,C), functional assays demonstrated that PLSCR1 depletion in dHL‐60 cells significantly reduced ROS levels and NET formation, as measured by DCFH‐DA probe staining (Figure 6K), and immunofluorescence (Figures 6L and S13D). qRT‐PCR analysis further showed that the mRNA levels of *CYBB* and *NCF1*, which encode components of the NADPH oxidase complex, were reduced in PLSCR1‐silenced dHL‐60 cells (Figure S13E). Importantly, immunofluorescence (Figures 6M and S14A) and Western blot analyses confirmed that re‐expression of PLSCR1 in METTL7A‐deficient dHL‐60 neutrophil‐like cells partially rescued NET formation (Figure S14B). These data support PLSCR1 as an important downstream effector that contributes to METTL7A‐dependent NET formation in vitro.

To explore potential m^6^A readers for PLSCR1 mRNA, we performed RIP‐qPCR using an anti‐YTHDF2 antibody, which showed enrichment of PLSCR1 transcripts in the YTHDF2 immunoprecipitate (Figure S14C). Furthermore, following YTHDF2 knockdown in dHL‐60 neutrophil‐like cells, whose efficiency was confirmed by qRT‐PCR at the mRNA level. YTHDF2 depletion led to reduced PLSCR1 mRNA levels (Figure S14D) and a shortened half‐life of PLSCR1 transcripts (Figure S14E). These observations suggest that YTHDF2 is associated with PLSCR1‐containing m^6^A‐regulated RNA complexes in this system. Because YTHDF2 can exert context‐dependent effects on RNA fate, additional work will be required to define the precise reader mechanism. Collectively, these findings support that METTL7A promotes neutrophil activation, functional maturation, ROS production, and NET formation at least in part through m^6^A‐dependent regulation of PLSCR1, a process that may contribute to the pathogenic role of neutrophils in psoriasis.

## 3 Discussion

This study elucidates a previously unrecognized pathway in which the m^6^A methyltransferase METTL7A governs neutrophil activation and effector function, specifically NETosis and ROS production, thereby driving psoriatic inflammation. Using an integrated approach combining patient‐derived neutrophils, transcriptomics, in vitro functional assays, and murine models, we demonstrate that METTL7A exerts its pathogenic role by stabilizing *PLSCR1* mRNA through m^6^A modification. Silencing or genetic deletion of *METTL7A* led to profound reductions in neutrophil maturation, NET formation, and proinflammatory cytokine release, resulting in significant amelioration of psoriasiform inflammation. Importantly, *METTL7A* expression was enriched in the CXCR4^hi^ neutrophil subset, a population we previously identified as proinflammatory and disease relevant in psoriasis. These findings position METTL7A as a pivotal upstream regulator of neutrophil heterogeneity and pathogenicity.

*Mettl7a* deficiency delayed spontaneous neutrophil apoptosis ex vivo but reduced neutrophil accumulation in inflamed skin in vivo. These findings are not contradictory, because tissue neutrophil burden is shaped by multiple processes, including recruitment, trafficking, activation, ROS production, and NET formation, rather than apoptosis alone [20]. Thus, the apoptosis data indicate that *METTL7A* influences neutrophil fate, whereas the net in vivo effect of *Mettl7a* loss reflects combined reductions in inflammatory recruitment and effector function.

Although m^6^A modifications have been increasingly recognized as critical regulators of cell differentiation and activity [21], prior studies have largely focused on canonical enzymes such as *METTL3* or *FTO* in lymphocytes [9, 22, 23] and macrophages [12, 24]. In psoriasis, m^6^A methylation mediated by *METTL3* in keratinocytes has been shown to regulates the mRNA of the lipid metabolic enzyme ELOVL6, thereby controlling neutrophil chemotaxis in skin inflammation [25]. In the context of neutrophil function, m^6^A dynamics have been implicated in regulating development, trafficking [13, 14], and inflammatory responses [26]. However, the contribution of nonclassical m^6^A writers to neutrophil biology, especially in chronic inflammatory diseases, has remained unexplored. Through RNA‐sequencing and bioinformatic analysis, *METTL7A* emerged as the most prominently upregulated m^6^A writer, distinguishing it from canonical enzymes like *METTL3* and *FTO*. Our study fills this gap by identifying *METTL7A* as a disease‐relevant m^6^A writer in neutrophils, thereby extending the epitranscriptomic paradigm beyond adaptive immunity into innate immune regulation. Compared with previously described transcription factors or signaling pathways, the METTL7A–PLSCR1 axis represents an epitranscriptomic checkpoint that directly links RNA modification with neutrophil effector functions, highlighting a novel layer of control over psoriatic inflammation.

Emerging evidence underscores METTL7A as a pivotal regulator in cell differentiation [15, 16] and physiological homeostasis [17]. In immune cells, *METTL7A* expression has been linked to enhanced immune cell infiltration and activation, often serving as a tumor suppressor by fostering T cell responses and antigen processing pathways in multiple human cancers [18]. Collectively, these insights position *METTL7A* as an indispensable orchestrator of both innate and adaptive immune dynamics, warranting further exploration of its therapeutic potential in autoimmune disorders like psoriasis, where dysregulated neutrophil activity predominates. By targeting *METTL7A*, interventions could achieve nuanced immune modulation, preserving protective responses while mitigating pathological inflammation, thus marking it as a cornerstone for advancing precision immunology.

Mechanistically, we found that *METTL7A* supports *PLSCR1* mRNA stability through m^6^A‐dependent regulation. PLSCR1 is a multifunctional immune regulator involved in membrane dynamics and inflammatory signaling. In neutrophils, PLSCR1 has been linked to surface exposure of proteinase 3 (PR3) and its interaction with antineutrophil cytoplasmic antibodies (c‐ANCA), thereby contributing to granulomatosis with polyangiitis [27]. Using methylated RNA immunoprecipitation, luciferase reporter assays, and mRNA stability analyses, our data support PLSCR1 as a downstream target of METTL7A‐dependent m^6^A regulation. Functionally, PLSCR1 depletion reduced ROS production and NET formation, whereas PLSCR1 re‐expression partially rescued impaired NET formation in *METTL7A*‐silenced cells. These findings suggest that the METTL7A–PLSCR1 axis contributes to neutrophil effector responses, although additional studies are needed to define the precise biochemical mechanism linking PLSCR1 to ROS generation and NET formation.

PLSCR1 is predominantly localized at the plasma membrane and contributes to membrane phospholipid asymmetry [28]. Through phosphatidylserine externalization, PLSCR1 has been linked to apoptosis [29] and phagocytosis [30]. Beyond these structural roles, PLSCR1 influences immune processes including mast cell degranulation [31] and antiviral defense [32, 33]. Studies focused on PLSCR1 in neutrophil biology remain limited. Current evidence indicates that PLSCR1 can enhance PR3–c‐ANCA binding on neutrophils and impair macrophage clearance of apoptotic neutrophils [27]. More recently, PLSCR1 expression has been associated with ROS‐related oxidative stress in red blood cells [34], and NET‐related PLSCR1 signatures have been linked to tumor biology [19]. Our data identify PLSCR1 as a functional downstream effector of METTL7A‐mediated m^6^A regulation in neutrophils. As a molecule connecting membrane dynamics, immune activation, and cell‐death‐associated processes, PLSCR1 warrants further investigation in inflammatory NET formation.

Several limitations should be acknowledged. First, larger, multicenter cohorts including diverse psoriasis subsets and treatment backgrounds are needed to validate the biomarker potential of METTL7A and PLSCR1. Second, although neutrophil depletion and adoptive transfer support a neutrophil‐intrinsic contribution of *Mettl7a*, these experiments cannot completely exclude contributions from residual host cells or other inflammatory compartments in vivo. Third, the current data link PLSCR1 to NADPH oxidase‐related gene expression and ROS output, but do not directly measure oxidase activation by subunit phosphorylation or membrane translocation. Fourth, although the *YTHDF2* data suggest reader involvement in *PLSCR1* mRNA regulation, other readers such as *YTHDF1*, *YTHDF3*, and *IGF2BP* family proteins were not systematically compared. Further mechanistic studies will be required to resolve these issues.

Through RIP‐qPCR and *YTHDF2* silencing experiments, we preliminarily identified *YTHDF2* as a potential m^6^A reader of *PLSCR1* mRNA, and our data suggest that *YTHDF2* primarily contributes to the maintenance of *PLSCR1* mRNA stability in our system. However, we did not systematically compare *YTHDF1*, *YTHDF3*, or other noncanonical readers such as *IGF2BPs*. Further mechanistic work, including competitive binding assays and CLIP‐seq, is required to fully define how METTL7A‐dependent m^6^A signals are decoded.

Therapeutically, targeting aberrant neutrophil activity remains a major challenge in chronic inflammatory and autoimmune diseases. Our discovery of the METTL7A–PLSCR1 axis provides a new avenue for therapeutic intervention. This pathway may extend beyond psoriasis to other NETosis‐associated diseases. By pinpointing METTL7A as a disease‐relevant m^6^A modifier in pathogenic neutrophils and identifying PLSCR1 as its downstream effector, our work offers a rationale for developing highly selective inhibitors targeting either METTL7A or its interaction with *PLSCR1* mRNA. However, the therapeutic feasibility and safety of such approaches require further validation, particularly because neutrophils are essential for host defense.

In summary, our work uncovers a novel mechanism whereby METTL7A, via m^6^A‐dependent regulation of *PLSCR1*, promotes neutrophil activation, ROS production, and NET formation, thereby fueling psoriatic inflammation. This study links epitranscriptomic regulation with pathogenic neutrophil programs and suggests METTL7A and PLSCR1 as potential biomarkers or therapeutic targets in neutrophil‐driven inflammatory diseases. Future efforts to pharmacologically inhibit *METTL7A* or disrupt its interaction with *PLSCR1* mRNA should determine whether pathogenic neutrophil responses can be selectively dampened while preserving essential host defense in psoriasis and related NETosis‐associated disorders.

## 4 Materials and Methods

### 4.1 Human Subjects

Peripheral blood and skin tissue samples from psoriasis patients were obtained from the Department of Dermatology, Xijing Hospital. The diagnosis of psoriasis was confirmed based on characteristic clinical and histologic features, with reference to established diagnostic criteria. Age and sex matched HC blood samples were collected from the Health Examination Center of Xijing Hospital. All HC individuals showed no evidence of chronic autoimmune diseases or acute infections. This study fully adhered to institutional regulations, and all procedures involving human materials were approved by the Ethical Committee of the Fourth Military Medical University and complied with the principles of the Declaration of Helsinki. Written informed consent was obtained from all participants.

### 4.2| m^6^A–RIP–qPCR Assay

The MeRIP–qPCR assay was conducted using a commercial methylated RNA immunoprecipitation kit (GS‐ET‐001; Cloudseq Biotech) following the manufacturer's protocol. Briefly, total RNA was isolated from neutrophil samples with TRIzol reagent (Invitrogen) and chemically fragmented using fragmentation buffer. The fragmented RNA was immunoprecipitated with either an anti‐m^6^A antibody or normal rabbit IgG as a control. The m^6^A–RNA complexes were then captured using Protein A/G magnetic beads and rigorously washed. After elution, the enriched RNA was purified, reverse‐transcribed, and subjected to qRT‐PCR amplification using the PrimeScript RT Kit (RR820A; Takara). Enrichment of target RNA was quantified as a percentage of input RNA.

The methods for blood sampling handling and neutrophil isolation, experimental animals, RNA m^6^A quantification, cell culture and reagents, cell transfection, RNA isolation and qRT‐PCR, RNA‐sequencing and bioinformatic analysis, immunofluorescence staining, Western blot analysis, flow cytometry analysis of mouse skin, predict m^6^A modification sites, phagocytosis assay, neutrophil function assay, NET formation assay, skin histopathology, ROS detection, dual‐luciferase reporter, ActD mRNA stability, and RIP (RNA Immuno‐precipitation) assay are provided in the Supporting Information: Materials and Methods.

### 4.2 Statistical Analysis

Statistical analyses were carried out with GraphPad Prism (version 9). Results are expressed as mean ± SEM. Comparisons between two groups were performed using two‐tailed paired or unpaired Student's *t*‐tests, while multigroup comparisons were analyzed by one‐way or two‐way ANOVA, followed by Tukey's posthoc test. Correlation analyses utilized Pearson's method for normally distributed data and Spearman's method for non‐normally distributed data. A *p* value less than 0.05 was considered statistically significant. All in vitro and in vivo experiments were repeated at least twice with consistent results.

## Author Contributions

W.T.L., J.K.L., and H.Y.Q. contributed to study design, data collection, and manuscript preparation. X.T., X.L., Y.X.B., K.L., and Z.G.L. performed data analysis and in vitro experiments. J.Q.W., Y.F.G., and X.L.S. contributed to patient sample collection. K.X. and E.L.D. revised the manuscript. J.L.C., G.W., and S.S. conceived the study, supervised the research, and critically revised the manuscript. All authors have read and approved the final manuscript.

## Funding

This work was supported by the National Natural Science Foundation of China (Grant No. 82322057, 82273520, and 82473516).

## Ethic Statement

All procedures involving human samples were approved by the Ethics Committee of the Xijing Hospital at the Fourth Military Medical University (Ethics Committee approval No: KY20203160‐1). All animal experiments were approved by the Animal Ethics Committee of the Fourth Military Medical University, Xi'an, China, under the approval number (SYXK(Shaanxi)‐2019‐001).

## Conflicts of Interest

The authors declare no conflicts of interest.

## Supporting information



Supporting File 01: mco270941‐sup‐0001‐SuppMat.docx

## Data Availability

The data underlying this article are available in the Genome Sequence Archive (GSA) [35, 36] at https://ngdc.cncb.ac.cn/gsa under accession numbers HRA005230, HRA013229, and CRA029900. The data will also be shared by the corresponding author upon reasonable request.
