# Supplementary material for: METTL7A‐Dependent m6A Methylation Orchestrates CXCR4hi Neutrophil Pathogenicity and NETosis in Skin Inflammation
Source: MedComm (2020). 2026 Aug 17;7(9):e70941. doi: 10.1002/mco2.70941 (PMC13482343; doi:10.1002/mco2.70941)
Supplement: Supplementary file 1 [file MCO2-7-e70941-s001.docx]

**Supplementary Materials for**

**METTL7A-Dependent m^6^A Methylation Orchestrates CXCR4^hi^ Neutrophil Pathogenicity and NETosis in skin inflammation**

**Running title:** METTL7A Orchestrates Neutrophil Pathogenicity

Wanting Liu, MS^#^, Jinkai Liang, MD^#^, Huiyi Quan, MD^#^, Xuan Liu, MM, Kang Li, MS, Xin Tang, MD, Jiaqi Wang, MM, Yunfeng Gu, MM, Yaxing Bai, MD, Ke Xue, PhD, Zhiguo Li, MD, Xiaolei Su, MM, Erle Dang, PhD, Jiaoling Chen, PhD^*^, Gang Wang, MD, PhD^*^, Shuai Shao, MD, PhD^*^

**Affiliations:**

Department of Dermatology, Xijing Hospital, Fourth Military Medical University, Xi’an, China

^#^Equally contributing authors

^*^**Corresponding author:**

Shuai Shao, shaos@fmmu.edu.cn

Gang Wang, [xjwgang@fmmu.edu.cn](mailto:xjwgang@fmmu.edu.cn)

Jiaoling Chen, [chenjiaoling1004@163.com](mailto:chenjiaoling1004@163.com)

**This file includes:**

Supplementary Materials and methods

Supplementary Figures:

Figure S1. METTL7A is upregulated in psoriatic neutrophils and shows spatialoverlap with NETosis-associated CitH3 signals in lesional skin.

Figure S2. METTL7A expression in neutrophils is associated with psoriasis activity, neutrophil maturation, and psoriatic serum stimulation.

Figure S3. Genotypic identification of METTL7A knockout mice by PCR.

Figure S4. Flow cytometric analysis of neutrophil infiltration in different tissues of WT and *Mettl7a^-/-^* mice after IMQ treatment.

Figure S5. Flow cytometric gating strategy and analysis of neutrophil distribution and activation after adoptive transfer.

Figure 6. Validation of METTL7A silencing efficiency and detection of METTL7A protein expression after interference.

Figure S7. Dynamics of m^6^A regulator expression in TNF-α-stimulated neutrophils.

Figure S8. METTL7A modulates neutrophil-like maturation, degranulation,and oxidative burst.

Figure S9. *Mettl7a* deficiency reduces ROS production and NET formation,delays spontaneous apoptosis, and alters aging-associated marker dynamics in primary mouse bone marrow neutrophils.

Figure S10. METTL7A silencing suppresses neutrophil activation- and inflammation-related transcriptional programs.

Figure S11. PLSCR1 expression is increased in psoriasis-associated neutrophils and during neutrophil differentiation.

Figure S12. The prediction of m^6^A modification sites on PLSCR1.

Figure S13. METTL7A stabilizes *PLSCR1* mRNA, and PLSCR1 silencing impairs NETosis-related responses in dHL-60 neutrophil-like cells.

Figure S14. PLSCR1 rescues impaired NET formation in METTL7A-deficient cells and is regulated by YTHDF2.

Supplementary Tables:

Table S1. Patient characteristics

Table S2. Primer list

Table S3. siRNA sequences list

Table S4. PLSCR1 m^6^A high-scoring prediction site primer sequence

**Supplementary Materials and Methods**

**Blood sampling handling and neutrophil isolation**

To eliminate the effects of circadian rhythms, all blood samples in this study were collected between 7:00 and 8:00 AM and immediately processed for cell isolation and serum collection. The isolated neutrophils were promptly used in subsequent experimental steps, while the serum was frozen at -80℃. Additionally, all selected participants reported regular work schedules and sleep-wake patterns over the previous 4 weeks.

For human neutrophil isolation, 4 mL of whole blood was carefully layered over 4 mL of Polymorphprep (1114683, Axis-Shield) in a 15 mL conical tube and centrifuged at 500 g and 20℃ for 30 minutes. The polymorphonuclear cell layer was collected, and residual red blood cells were lysed using RBC lysis buffer (FXP001, 4A Biotech Co.). Isolated neutrophils were resuspended in PBS at a concentration of 1×10^7^ cells/mL.

To isolate CXCR4^hi^ neutrophils, magnetically labeled cells were retained in a magnetic field using a MACS separator (130-090-312, Miltenyi Biotec). Briefly, neutrophils were resuspended in 100 μL MACS buffer (130-091-221, Miltenyi Biotec), stained with APC-conjugated anti-human CXCR4 antibody (2 μL/10^7^ cells) for 10 minutes, washed, and incubated with anti-APC MicroBeads (20 μL/10^7^ cells, 130-100-070, Miltenyi Biotec) in 80 µL separation buffer for 15 minutes in the dark. The cell suspension was then applied to a pre-washed MS column (130-042-201) placed in the separator. Unlabeled cells passed through, while labeled CXCR4^hi^ neutrophils were retained and eluted after removal from the magnetic field.

For mouse neutrophil isolation, bone marrow cells were harvested from femurs of mice anesthetized with intraperitoneal 1% sodium pentobarbital (4579, 100-150 μL/mouse, R&D Systems). And peripheral blood was pooled from five mice per isolation. Erythrocytes were lysed using RBC lysis buffer (FXP001, 4A Biotech Co.). Up to 1×10^8^ cells were suspended in 200 µL MACS buffer (130-091-221, Miltenyi Biotec), incubated with 50 µL anti-Ly6G Biotin MicroBeads (130-120-337, Miltenyi Biotec) per sample for 15 minutes at 4℃ in the dark, washed, and applied to an LS column (130-042-401) placed in a MACS separator (130-042-303). The Ly6G^+^ neutrophils were retained on the column, eluted, washed, and centrifuged at 300 × g for 10 minutes before resuspension in PBS for subsequent use.

**Experimental animals**

C57BL/6J mice (8-10 weeks old) were purchased from the Department of Laboratory Animal Medicine, Fourth Military Medical University (Xi'an, Shaanxi, China). *Mettl7a* knockout mice (*Mettl7a*^-/-^, 8-10 weeks old) in the C57BL/6 background were purchased from the Cyan Biosciences (Suzhou, China). Genotyping of the offspring mice was performed using *Mettl7a*^-/-^ primers: F1, 5’-GGGTTTCCTTTCAC TTCCTTTACA-3’; R1, 5’-AGACCAGGCTGTACTCAAGGAT-3’; wild-type (WT) primers: F2, 5’-AGTGGCTCTCTGTCCTTTGGCT-3’; R1, 5’-AGACCAGGCTGTACTCAAGGAT-3’. All experimental procedures involving mice were approved by the institutional animal care and use committee of the Fourth Military Medical University and complied with National Institutes of Health Guidelines for the Humane Treatment of Laboratory Animals.

All experiments utilized both male and female mice. Animals were randomly assigned to experimental groups (n = 6 per group) and maintained under specific pathogen-free barrier conditions. For *in vivo* studies, investigators were blinded to treatment groups until all data analyses were completed. At the endpoint, mice were humanely euthanized via sodium pentobarbital overdose. To establish a psoriasis-like mouse model, a daily topical dose of 62.5 mg IMQ cream (5% IMQ; iNova Pharmaceuticals) was applied to the shaved dorsal skin of mice for five consecutive days. Neutrophil depletion was achieved through intraperitoneal injection of either purified anti-Ly6G antibody (127649, BioLegend) or an isotype control antibody (400565, BioLegend) administered every other day, starting with an initial dose of 100 μg followed by 50 μg for subsequent injections. In some experiments, IMQ-treated mice received subcutaneous injections of purified *Mettl7a*^-/-^ or WT neutrophils (6 × 10^5^ cells per mouse) isolated from the peripheral blood of syngeneic donor mice. A detailed schematic of the experimental timeline is provided in the supplementary materials.

Peripheral blood was collected from *Mettl7a*^-/-^ and WT mice, and neutrophils were isolated using mouse Ly6G MicroBeads (130-120-337, Miltenyi Biotec) in accordance with the manufacturer’s instructions.

**RNA m^6^A quantification**

Total RNA was isolated with TRIzol reagent following the manufacturer’s protocol. Global m^6^A levels in the extracted RNA were measured using the EpiQuick™ m^6^A RNA Methylation Quantification Kit (Colorimetric) (P-9005, EpigenTek). For each assay, 200 ng of total RNA was loaded per well. After thorough washing, a capture antibody was applied, followed by sequential incubation with detection antibody, enhancer solution, and color development solution to initiate chromogenesis, according to the kit instructions. The m^6^A content was quantified by measuring absorbance at 450 nm with reference to a standard curve.

**Cell culture and reagents**

The HL-60 cell line (CL-0110) was purchased from Procell (Wuhan, China) and maintained in complete medium under recommended conditions. To induce neutrophil-like differentiation (dHL-60), HL-60 cells were treated with 1.25% DMSO and cultured in a CO_2_ incubator for 5 days, yielding dHL-60 cells. Cellular morphology was examined under a microscope to confirm differentiation. For stimulation experiments, dHL-60 cells were treated with vehicle, recombinant human TNF-α (50 ng/mL), or serum from healthy controls (HC) or psoriasis patients (PSO) for 24 h. All cell lines were confirmed negative for mycoplasma contamination.

**Cell transfection**

Synthetic double-stranded small interfering RNA (siRNA) targeting human *METTL7A* ,*PLSCR1* and *YTHDF2* was purchased from Biopell biotechnology. The siRNA sequences used are provided in Supplementary Table 3. dHL-60 cells were seeded into 6-well plates at a density of 5 ×10^5^ cells/well and transfected with siRNA using Lipofectamine 2000 transfection reagent (MF135-01, Mei5 Biotechnology) according to the manufacturer’s instructions.

**RNA isolation and qRT-PCR**

RNA was isolated using TRIzol reagent (15596018CN, Invitrogen) according to the manufacturer's protocol. The concentration and purity of the extracted RNA were assessed with a spectrophotometer (N12391, Thermo Fisher Scientific). Quantitative real-time PCR (qRT-PCR) was conducted in 384-well plates with SYBR Green Master Mix (RR820A, TaKaRa), following the supplier's instructions. mRNA expression levels were normalized to β-actin as the internal reference gene and calculated using the 2^−ΔΔCt^ method. All primer sequences used in the qRT-PCR assays are listed in Supplementary Table 2.

**RNA-sequencing and bioinformatic analysis**

Total RNA was isolated from dHL-60 cells representing four experimental groups: siNC, siMETTL7A, TNF-α+siNC and TNF-α+siMETTL7A. Mouse neutrophils were purified from bone marrow using Ly6G^+^ magnetic beads, corresponding to the following groups: WT + vehicle, *Mettl7a^-/-^* + vehicle, WT+IMQ and *Mettl7a^-/-^* + IMQ. RNA extraction was carried out with TRIzol reagent (15596018CN, Invitrogen) in strict accordance with the manufacturer's instructions. The resulting RNA samples were submitted to Majorbio for RNA sequencing. The sequencing procedure included the following steps: quality assessment of total RNA, mRNA enrichment, double-stranded cDNA synthesis, end repair/dA-tailing, fragment selection (3’ end), PCR amplification, library quality control, and Illumina sequencing. Differentially expressed genes were defined using a threshold of FDR < 0.05 and an fold change > 1.5. The raw sequencing data have been deposited in the Genome Sequence Archive at the National Genomics Data Center, Beijing Institute of Genomics, Chinese Academy of Sciences, under accession number CRA029900 (mouse neutrophils) and HRA013229 (dHL-60 cells).

**Immunofluorescence staining**

For tissue samples, 4μm-thick paraffin-embedded sections were dewaxed and rehydrated. Cultured dHL-60 cells grown on cover slides were fixed with 4% paraformaldehyde for 15 minutes. After blocking with goat serum for 1 hour at room temperature, both tissue sections and cells were incubated with primary antibodies at 4℃ overnight. The following primary antibodies were used: rat anti-Ly6G monoclonal antibody (SC-53515, Santa; 1:100), mouse anti-CD15 monoclonal antibody (241552, Abcam; 1:100), rabbit anti-CD15 monoclonal antibody (135377, Abcam; 1:100), rabbit anti-CXCR4 monoclonal antibody (18102, Abcam; 1:100), mouse anti-CXCR4 monoclonal antibody (60042-1-1g, Proteintech; 1:100), goat anti-CXCR4 polyclonal antibody (GTX21671, GeneTex; 1:100), and anti-METTL7A antibody. Samples were washed three times with PBS and then incubated with corresponding fluorophore-conjugated secondary antibodies for 1 hour at room temperature, followed by three 10 minutes washes with PBS. Nuclei were stained with Hoechst 33258 (C0021, Solarbio; 1:1000), and images were acquired using a confocal microscope (LSM 880, Carl Zeiss).

For neutrophil immunofluorescence, cells were fixed, permeabilized, and processed as described above. The primary antibodies applied included: mouse anti-CD15 (241552, Abcam; 1:100), rabbit anti-CD15 (135377, Abcam; 1:100), mouse anti-CXCR4 (60042-1-1, Proteintech; 1:100), goat anti-CXCR4 (GTX21671, GeneTex; 1:100), and rabbit anti-METTL7A polyclonal antibody (GTX65969, GeneTex; 1:100). Cells were resuspended in 100 μL PBS, placed on cover slides, and incubated overnight prior to imaging with the LSM 880 confocal microscope.

**Western blot analysis**

As previously mentioned, the total protein was extracted from the cells using radioimmunoprecipitation assay (RIPA) lysis buffer. Protein concentrations were measured with BCA protein assay kit (PA115-02, TIANGEN). The equivalent protein was then isolated on a 10% gel by SDS-polyacrylamide gel electrophoresis and transferred to a polyvinylidene fluoride membrane (Invitrogen, Carlsbad). After being sealed with a blocking buffer for 2 hours, the membrane was treated with Mouse monoclonal to GAPDH (60004-1-Ig, 1:5000, Proteintech), Mouse monoclonal antibody to PADI4 (128086, 1:1000, Abcam), rabbit polyclonal antibody to citrullinated histone-3 (5103, 1:1000, Abcam), mouse monoclonal antibodies to CXCR4 (60042-1-IG, 1:1000, Proteintech), METTL7A Polyclonal antibody (17-92-1-AP, 1:5000, Proteintech) and PLSCR1 Rabbit mAb (A3430, 1:1000, ABclonal) were kept overnight at 4 °C. The membrane was then incubated at room temperature for 1 hour with a secondary antibody conjugated to either rabbit or mouse horseradish peroxidase. The blot was detected using an enhanced chemiluminescence assay kit (GTX 14698, GeneTex). Via Image Lab (Bio-Rad Laboratories, Inc., version 5.2.1). Uncropped and unprocessed scans are provided in source data files and supplementary information.

**Flow cytometry analysis of mouse skin**

For flow cytometry, a 1 cm×1 cm piece of dorsal mouse skin was dissected and transferred to a 1.5 mL EP tube containing 1 mL of Hank’s Balanced Salt Solution (HBSS; H4641, Sigma-Aldrich). The tissue was washed thoroughly by vigorous vortexing for 15 seconds, repeated three times.

The skin was then minced into fragments smaller than 0.5 mm in a pre-chilled 6-well plate using unsupplemented Dulbecco’s Modified Eagle Medium (DMEM; 11885-084, Gibco) enriched with 1 mg/mL Collagenase P (11213857001, Roche) and 0.2 mg/mL DNase I (AMPD1, Sigma-Aldrich). Digestion was carried out for 60 minutes at 37 ℃ in a cell culture incubator, with gentle pipetting every 20 minutes to promote tissue dissociation.

The digested cell suspension was filtered through a 40 μm strainer. Cells were stained for 30 minutes in FACS buffer using the following antibodies: PerCP/Cy5.5 anti-mouse CD45 (103132; 1:100, BioLegend), FITC anti-mouse Ly6G (127606; 1:100, BioLegend), PE anti-mouse CXCR4 (146506; 1:100, BioLegend), and Zombie UV™ viability dye (423102; 1:500, BioLegend). Data were acquired on a BD LSRFortessa™ cell analyzer (649225) and analyzed with FlowJo v10.8.1 (Tree Star). Background fluorescence was evaluated using fluorescence-minus-one (FMO) controls.

**Predict of m^6^A modification sites**

The Sequence-based RNA Adenosine Methylation site Predictor (SRAMP) was used to predict m^6^A modification sites on PLSCR1 RNA sequences. Based on the high-confidence m^6^A prediction results, specific qRT-PCR primers were designed (see Table S4).

**Phagocytosis assay**

To evaluate phagocytic capacity, dHL-60 cells transfected with siNC or siMETTL7A were incubated with pHrodo™ Green E. coli BioParticles® (2 mg/mL; P35366, Thermo Fisher Scientific, USA) at 37℃ for 30 minutes. The incubation was terminated by adding 2 mL of PBS, followed by three washes with PBS. Phagocytic uptake was quantified using flow cytometry and expressed as median fluorescence intensity (MFI).

**Neutrophil function assay**

Neutrophil degranulation was evaluated by measuring the cell surface expression of CD63. dHL-60 cells transfected with siNC or siMETTL7A were stimulated with either solvent or TNF-α for 4 hours. After harvesting, the cells were washed twice with PBS and stained in the dark at 4℃ for 30 minutes using the following fluorescently labeled antibodies: FITC mouse anti-human CD15 (301904, 1:100, BioLegend), PE-Cy7 mouse anti-human CXCR4 (306514, 1:100, BioLegend), and PE mouse anti-human CD63 (353004, 1:100, BioLegend). After three washes with PBS, the cells were resuspended and acquired on a BD LSRFortessa flow cytometer (649225). Background fluorescence was determined using fluorescence minus one (FMO) controls.

**NET formation assay**

For isolation of primary mouse bone marrow neutrophils, bone marrow cells were flushed from femurs and tibiae of mice. After red blood cell lysis, neutrophils were purified using anti-Ly6G microbeads (130-120-337, Miltenyi Biotec) according to the manufacturer’s instructions. To evaluate NET formation, bone marrow Ly6G^+^ neutrophils or dHL-60 cells transfected with non-targeting control siRNA (siNC) or *METTL7A*-targeting siRNA (siMETTL7A) were seeded onto poly-D-lysine-coated coverslips (P4832, Sigma-Aldrich) at a density of 10^5^ cells per well, stimulated with PMA (100 nM, 524400, Sigma), and cultured at 37℃ for 4 hours. Cells were then fixed with 4% paraformaldehyde. After blocking with 3% bovine serum albumin in PBS for 2 hours at room temperature, the cells were incubated overnight at 4℃ with the following antibodies: rabbit anti-citrullinated histone H3 (CitH3; ab5103, Abcam; 1:200 dilution) and mouse anti-myeloperoxidase (MPO; ab25989, Abcam; 1:100 dilution). In parallel, SYTOX Green (150nM, 1S7020, Thermo Fisher) was used to visualize extracellular DNA.NET formation was quantified primarily by extracellular DNA staining and web-like chromatin morphology. Subsequently, cells were stained with Cy3-conjugated goat anti-mouse IgG (ab97035, Abcam; 1:1000) and Alexa Fluor 488-conjugated goat anti-rabbit IgG (ab150077, Abcam; 1:100). Following washes, nuclei were counterstained with Hoechst 33258 (C0021, SolarBio; 1:1000) and imaged using a confocal microscope (LSM 880, Carl Zeiss). NET formation was quantified primarily by SYTOX Green‑positive extracellular DNA structures with web‑like morphology. At least five randomly selected fields per sample were analyzed. CitH3 and extracellular MPO staining were used as supportive markers rather than stand‑alone quantitative readouts.

**Skin histopathology**

Skin tissue specimens were fixed, paraffin-embedded, and sectioned at 4 μm thickness for hematoxylin and eosin (H&E) staining. Slides were digitally scanned using a slide scanner and analyzed with NDP.view2 software (Hamamatsu Photonics). The dermal vascular area in H&E-stained sections was quantitatively assessed.

**ROS detection**

To detect intracellular ROS, dHL-60 cells were transfected with siRNA and subsequently treated with 100 nM phorbol 12-myristate 13-acetate (PMA; P1585, Sigma-Aldrich) or TNF-α at 37℃ for 30 minutes. The cells were then stained with a FITC-conjugated anti-human CD15 antibody (301904, 1:100, BioLegend). Intracellular ROS levels were assessed using a DCFH-DA probe kit (BB-47051, BestBio) at 37℃. After washing, flow cytometric analysis was performed immediately.

**Dual-luciferase reporter**

The wild-type (WT) or mutant (MUT) 3’UTR fragment of PLSCR1 containing the predicted m6A motif was cloned into the pmirGLO vector (Promega) downstream of the firefly luciferase gene. For reporter assays, dHL-60 cells cultured in 6-well plates were first transfected with a mixture containing 2.5 μg of the WT or MUT reporter plasmid and 50 ng of the Renilla luciferase internal control plasmid (pRL-TK, Promega) using Lipofectamine 3000 and P3000 reagents (L3000075, Invitrogen). Six hours post-transfection, the medium was replaced, and the cells were then transfected with either 2.5 μg of control vector or METTL7A overexpression vector (OE‑METTL7A). At 48 h after the second transfection, firefly and Renilla luciferase activities were measured sequentially using the Dual-Luciferase Reporter Assay System (JKR23008, GENECREATE) on a luminometer (Tecan i-control). Relative luciferase activity was calculated as the ratio of firefly to Renilla luminescence and normalized to the control group. Each condition was tested in triplicate, and three independent experiments were performed.

**ActD mRNA stability**

dHL-60 cells transfected with siNC, siMETTL7A, or siYTHDF2 were treated with actinomycin D (ActD, HY-17559, MCE) to block de novo transcription. Total RNA was extracted using TRIzol reagent (Invitrogen) at 0, 0.5, 1, and 2 h after ActD addition. *PLSCR1* mRNA levels were measured by qRT‑PCR and normalized to the internal control GAPDH. The remaining mRNA fraction at each time point was calculated relative to the 0 h time point. The mRNA decay curve was plotted, and the half‑life was determined by nonlinear regression using a one‑phase exponential decay model (GraphPad Prism). Three independent experiments were performed, each with three technical replicates.

**Apoptosis and flow cytometry analysis**

Freshly isolated Ly6G^+^ bone marrow neutrophils from wild-type (WT) and *Mettl7a^⁻/⁻^* mice were cultured in vitro for 0, 24, or 48 h as indicated. For apoptosis analysis, cells were stained with Annexin V and 7‑AAD using an Annexin V-APC/7‑AAD Apoptosis Detection Kit (AP105, MULTI SCIENCES) according to the manufacturer’s instructions, and analyzed by flow cytometry. Early and late apoptotic populations were quantified using FlowJo software. For aging‑marker analysis, cells were stained with PE anti-mouse CXCR4 (146506; 1:100, BioLegend), PE-cy7 anti-mouse CD101 (25-1011-82,1:100, ThermoFisher), and BV650 anti-mouse CD62L (104453, 1:100, BioLegend), neutrophils were analyzed at each time point by flow cytometry.

**RIP (RNA Immunoprecipitation) assay**

dHL‑60 cells were crosslinked with 1% formaldehyde for 10 min at room temperature, and the reaction was quenched with 0.125 M glycine. Cells were lysed in RIP lysis buffer (GS-ET-006, Genseq). Lysates were sonicated and clarified by centrifugation. For immunoprecipitation, 2 μg of anti‑YTHDF2 antibody (or control IgG) was incubated with protein A/G magnetic beads at 4 °C for 2 h, then mixed with the cleared lysate and incubated overnight at 4 °C. A portion of the lysate (10%) was saved as input control. After extensive washing with RIP wash buffer, the immunoprecipitated complexes were resuspended in elution buffer containing proteinase K (0.5 mg/mL) and incubated at 65 °C for 30 min to reverse crosslinks and digest proteins. RNA was extracted using TRIzol reagent and analyzed by qRT‑PCR with specific primers targeting the PLSCR1 (see Table S4). Enrichment was calculated as fold change relative to the IgG control using the 2⁻^ΔΔCt^ method. Three independent experiments were performed.

**PLSCR1 rescue experiments**

For METTL7A silencing, dHL-60 cells were transfected with siMETTL7A or siNC for 24 h. For rescue experiments, 24 h after siMETTL7A transfection, cells were further transfected with a PLSCR1 overexpression plasmid (pcDNA3.1‑PLSCR1) or an empty vector (pcDNA3.1) using Lipofectamine 3000 (L3000075, Invitrogen). At 48 h after the second transfection, cells were harvested for functional assays. Each condition was tested in at least three independent experiments.

**Supplementary Figure legends**

**Figure** S**1.METTL7A is upregulated in psoriatic neutrophils and shows spatialoverlap with NETosis-associated CitH3 signals in lesional skin.** (**A**) Volcano plots of methyltransferase genes in CXCR4^hi^ and CXCR4^lo^ neutrophils from healthy controls (HC) and psoriasis (Pso) patients. (**B**) Relative mRNA expression of m^6^A modification regulators (*METTL3*, *METTL4*, *METTL5*, *METTL14*, *WTAP*, *FTO*, *YTHDF1*, *YTHDF2*) in neutrophils from healthy controls (HC) and patients with psoriasis (Pso). (**C**) Representative immunofluorescence images of psoriatic lesional skin showing the distribution of CD15, METTL7A, and CXCR4. Scale bar, 100 μm. (**D**) Representative immunofluorescence images of skin sections from healthy controls (HC) and patients with psoriasis (Pso) showing Hoechst-stained nuclei (blue), CitH3 (green), and METTL7A (red). Scale bars, 100 μm. (**E**) Relative mRNA expression of METTL family members (*METTL2A*, *METTL9*, *METTL5*, *METTL17*, and *METTL23*) in CXCR4^lo^ and CXCR4^hi^ neutrophils from healthy controls (HC) and patients with psoriasis (Pso). Data are presented as mean ± SD. *^*^P* < 0.05, *^**^P* < 0.01; *ns*, not significant.

**Figure** S**2. METTL7A expression in neutrophils is associated with psoriasis activity, neutrophil maturation, and psoriatic serum stimulation. (A)** Relative *METTL7A* mRNA expression in peripheral blood neutrophils from healthy controls (HC) and patients with psoriasis (Pso), and paired post-treatment samples from the same psoriasis patients receiving biologic therapy (post-treatment) (n = 10). **(B)** Representative Western blot and quantification of METTL7A protein levels in neutrophils from healthy controls (HC) and patients with psoriasis (Pso), and paired post-treatment samples from the same psoriasis patients receiving biologic therapy (post-treatment). TUBULIN was used as the loading control. **(C)** Global m^6^A modification levels in total RNA from neutrophils of patients with psoriasis (Pso), and paired post-treatment samples from the same psoriasis patients receiving biologic therapy (post-treatment) (n = 10). **(D)** Heatmap of the relative expression of METTL7A and CD101 in CXCR4^lo^ and CXCR4^hi^ neutrophil subsets. **(E)** Relative *METTL7A* mRNA expression in dHL-60 neutrophil-like cells stimulated with serum from healthy controls (HC) and patients with psoriasis (Pso). **(F)** Representative Western blot and quantification of METTL7A protein levels in dHL-60 neutrophil-like cells stimulated with serum from healthy controls (HC) or patients with psoriasis (Pso). TUBULIN was used as the loading control. Data are presented as mean ± SD. *^*^P* < 0.05, *^**^P* < 0.01, *^***^P* < 0.001.

**Figure** S**3. Genotypic identification of METTL7A knockout mice by PCR.** **(A)** Genomic DNA was extracted from mouse tail tissue and amplified by PCR using Mettl7a-specific primers. The PCR products were separated by electrophoresis on a 2% agarose gel. Lane M: DNA molecular weight marker (DL2000). Lanes 1-5: Homozygous mutant mice showing a single band at 323 bp. Lanes 6-7: Heterozygous mice showing two bands at 323 bp and 449 bp. Lanes 8-9: Negative control (ddH_2_O)

**Figure** S**4. Flow cytometric analysis of neutrophil infiltration in different tissues of WT and *Mettl7a^-/-^* mice after IMQ treatment.** **(A)** Representative gating strategy, flow plots and quantification of CD11b^+^Ly6G^+^ neutrophils in bone marrow from WT and *Mettl7a*^-/-^ mice treated with vehicle or IMQ (n = 6). **(B)** Representative flow plots and quantification of CD11b^+^Ly6G^+^ neutrophils in spleen under indicated conditions (n = 6). Statistical significance was determined by two-way ANOVA where appropriate. Data are presented as mean ± SEM. ^***^*P* < 0.001 and *ns,* not significant.

**Figure** S**5. Flow cytometric gating strategy and analysis of neutrophil distribution and activation after adoptive transfer.** **(A)** Gating strategy for Single LIVE CD45^+^ cells (representative plots). **(B)** Representative flow plots showing CD11b^+^Ly6G^+^ cell proportions in skin (upper row), bone marrow (middle row), and spleen (lower row) across indicated groups. **(C)** Representative histograms of CXCR4 expression in these neutrophils across indicated groups.

**Figure** S**6. Validation of METTL7A silencing efficiency and detection of METTL7A protein expression after interference. (A)** Western blot analysis of METTL7A protein levels in dHL-60 neutrophil-like cells transfected with siNC or three independent siRNAs targeting METTL7A (siMETTL7A-1/2/3). TUBULIN served as the loading control. **(B)** Quantification of *METTL7A* mRNA expression in dHL-60 neutrophil-like cells transfected with siNC or three independent siRNAs targeting METTL7A (siMETTL7A-1/2/3) (n = 6). **(C)** Western blot analysis of METTL7A protein levels in siNC- or siMETTL7A-transfected dHL-60 neutrophil-like cells with or without TNF-α stimulation. GAPDH served as the loading control. Statistical significance was determined by one-way ANOVA where appropriate. Data are presented as mean ± SEM. *^***^P* < 0.001.

**Figure** S**7. Dynamics of m^6^A regulator expression in TNF**-α **- stimulated neutrophils. (A)**Time-course of relative mRNA expression of *METTL7A*, *METTL3*, *METTL14*, *FTO*, and *WTAP* in dHL-60 neutrophil-like cells stimulated with TNF-α for 0-8 h. Statistical significance was determined by unpaired Student’s t-test or one-way ANOVA where appropriate. Data are presented as mean ± SEM. *^*^P* < 0.05, *^**^P* < 0.01, *^***^P* < 0.001 and *ns*, not significant.

**Figure** S**8. METTL7A modulates neutrophil-like maturation, degranulation,and oxidative burst. (A)** Representative gating strategy and flow cytometry histograms of CXCR4, CD101, CD66b, CD63 in dHL-60 neutrophil-like cells transfected with control siNC or siMETTL7A, with or without TNF-α stimulation. **(B)** Representative gating strategy and flow cytometry histograms of ROS production in dHL-60 neutrophil-like cells transfected with control siNC or siMETTL7A, with TNF-α or PMA stimulation.

**Figure** S**9. *Mettl7a* deficiency reduces ROS production and NET formation,delays spontaneous apoptosis, and alters aging-associated marker dynamics in primary mouse bone marrow neutrophils. (A)** Representative flow cytometry gating strategy for the isolation and identification of primary bone marrow neutrophils (CD11b^+^Ly6G^+^) from wild-type (WT) and *Mettl7a*^-/-^ mice. **(B)** Representative flow cytometry histograms and quantification of intracellular ROS levels in wild-type (WT) and *Mettl7a*^-/-^ bone marrow neutrophils treated with vehicle or PMA. **(C)** Representative immunofluorescence images and quantification of NET formation in wild-type (WT) and *Mettl7a*^-/-^ neutrophils treated with vehicle or PMA. Scale bar, 100 μm. **(D)** Representative flow cytometry plots of Annexin V/7-AAD staining and quantification of early and late apoptotic rates in wild-type (WT) and *Mettl7a*^-/-^ neutrophils after 48 h of *in vitro* culture (n = 6). **(E)** Flow cytometry quantification of the aging- and migration-associated surface markers CXCR4, CD101, and CD62L in wild-type (WT) and *Mettl7a*^-/-^ neutrophils at 0, 24, and 48 h of *in vitro* culture. Data are presented as mean ± SD. *^**^P* < 0.01, *^***^P* < 0.001; *ns*, not significant.

**Figure** S**10. *METTL7A* silencing suppresses neutrophil activation- and inflammation-related transcriptional programs. (A)** GO and KEGG pathway enrichment analysis of DEGs in dHL-60 neutrophil-like cells transfected with siNC versus siMETTL7A under basal conditions. Data are shown as fold enrichment with corresponding P values; bubble size indicates gene count.

**Figure** S**11. PLSCR1 expression is increased in psoriasis- associated neutrophils and during neutrophil differentiation.** **(A)** Western blot analysis of PLSCR1 protein expression in neutrophils from healthy controls (HC) and patients with psoriasis (Pso). GAPDH served as the loading control. **(B** and **C)** qRT-PCR and Western blot analysis of PLSCR1 expression in neutrophils from healthy controls (HC) and patients with psoriasis (Pso), and paired post-treatment samples from the same psoriasis patients receiving biologic therapy (post-treatment). TUBULIN served as the loading control. **(D** and **E)** qRT-PCR and Western blot analysis of PLSCR1 expression in dHL-60 neutrophil-like cells stimulated with serum from healthy controls (HC) and patients with psoriasis (Pso). TUBULIN served as the loading control. **(F** and **G)** qRT-PCR and Western blot analysis of PLSCR1 expression during DMSO-induced HL-60 neutrophil differentiation. TUBULIN served as the loading control. **(H)** Western blot analysis of PLSCR1 protein levels in dHL-60 neutrophil-like cells transfected with siNC or siMETTL7A, with or without TNF-α stimulation .Data are presented as mean ± SD. *^*^P* < 0.05, *^**^P* < 0.01, *^***^P* < 0.001.

**Figure** S**12. Prediction of m^6^A modification sites on PLSCR1.**

**(A)** The m^6^A modification of mRNA sequence of *PLSCR1* [Homo sapiens (human)] in the SRAMP prediction servers. A very high confidence m^6^A sites at position 944 in the coding sequence (CDS) region.

**Figure** S**13. METTL7A stabilizes *PLSCR1* mRNA, and PLSCR1 silencing impairs NETosis-related responses in dHL-60 neutrophil-like cells. (A)** mRNA stability analysis of *PLSCR1* in siNC and siMETTL7A-transfected dHL-60 neutrophil-like cells after transcriptional inhibition (n = 6). **(B** and **C)** qRT-PCR and Western blot analysis of PLSCR1-silencing efficiency in dHL-60 neutrophil-like cells transfected with siNC or indicated siPLSCR1 sequences. **(D)** Representative immunofluorescence images and quantification of NET formation in PMA-stimulated dHL-60 neutrophil-like cells transfected with siNC or siPLSCR1, stained for MPO and CitH3. **(E)** qRT-PCR analysis of *CYBB* and *NCF1* mRNA expression in siNC and siPLSCR1-transfected dHL-60 neutrophil-like cells (n = 3). Data are presented as mean ± SD. *^***^P* < 0.001.

**Figure** S**14. PLSCR1 re-expression partially rescues impaired NET formation inMETTL7A-deficient cells, and YTHDF2 is associated with PLSCR1 RNA stability. (A)** Representative immunofluorescence images and quantification of NET formation in PMA-stimulated METTL7A-silenced dHL-60 neutrophil-like cells with or without PLSCR1 re-expression, stained for MPO and CitH3. **(B)** Western blot analysis of PAD4 and CitH3 expression in PMA-stimulated dHL-60 neutrophil-like cells transfected with siNC or siMETTL7A and re-transfected with Control Vector or PLSCR1 overexpression vector (OE-PLSCR1). TUBULIN served as the loading control. **(C)** RIP-qPCR analysis of PLSCR1 enrichment in YTHDF2 immunoprecipitates compared with IgG control (n = 6). **(D)** qRT-PCR analysis of YTHDF2 silenced efficiency in dHL-60 neutrophil-like cells transfected with siNC or indicated siYTHDF2 sequences (n = 3). **(E)** mRNA stability analysis of *PLSCR1* in siNC- and siYTHDF2-transfected dHL-60 neutrophil-like cells after transcriptional inhibition (n = 6). Data are presented as mean ± SD. *^*^P* < 0.05, *^***^P* < 0.001

**Supplementary Tables**

| **Number** | **Sex** | **Age(years)** | **PASI** | **Comorbidities** |
| --- | --- | --- | --- | --- |
| Pso-1 | Female | 23 | 14.4 | No |
| Pso-2 | Male | 39 | 17.6 | No |
| Pso-3 | Male | 37 | 11.2 | No |
| Pso-4 | Male | 27 | 21.9 | No |
| Pso-5 | Male | 33 | 14.4 | No |
| Pso-6 | Female | 24 | 16.7 | No |
| Pso-7 | Female | 37 | 13.1 | No |
| Pso-8 | Female | 33 | 17 | No |
| Pso-9 | Male | 32 | 11.5 | No |
| Pso-10 | Male | 27 | 17.8 | No |
| Pso-11 | Female | 47 | 8.4 | No |
| Pso-12 | Female | 31 | 12.5 | No |
| Pso-13 | Male | 32 | 13.5 | No |
| Pso-14 | Male | 46 | 18.7 | No |
| Pso-15 | Male | 32 | 10.4 | No |
| Pso-16 | Female | 40 | 11.4 | No |
| Pso-17 | Male | 38 | 18.7 | No |
| Pso-18 | Male | 21 | 12.3 | No |
| Pso-19 | Female | 41 | 8.4 | No |
| Pso-20 | Female | 32 | 10.8 | No |
| Pso-21 | Male | 24 | 16.2 | No |
| Pso-22 | Male | 26 | 28.6 | No |
| Pso-23 | Male | 24 | 30 | No |
| Pso-24 | Female | 45 | 10.9 | No |
| Pso-25 | Male | 33 | 20 | No |
| Pso-26 | Male | 22 | 10.2 | No |
| Pso-27 | Female | 56 | 10.4 | No |
| Pso-28 | Female | 19 | 14.8 | No |
| Pso-29 | Male | 29 | 17.6 | No |
| Pso-30 | Male | 27 | 13.4 | No |
| Pso-31 | Male | 32 | 15 | No |
| Pso-32 | Female | 50 | 19.2 | No |
| Pso-33 | Male | 35 | 14.9 | No |
| Pso-34 | Male | 39 | 14.8 | No |
| Pso-35 | Male | 25 | 19.2 | No |

**Table S1. Patient characteristics**

**Table S2. Primer list**

| **Primer** | **sequences** |
| --- | --- |
| Human  IL-17A | Forward 5’- CGGACTGTGATGGTCAACCTGA -3’ |
|  | Reverse 5’- GCACTTTGCCTCCCAGATCACA -3’ |
| Human  IL-6 | Forward 5’- AGACAGCCACTCACCTCTTCAG -3’ |
|  | Reverse 5’- TTCTGCCAGTGCCTCTTTGCTG -3’ |
| Human  LCN2 | Forward 5’- GTGAGCACCAACTACAACCAGC -3’ |
|  | Reverse 5’- GTTCCGAAGTCAGCTCCTTGGT -3’ |
| Human  LL37 | Forward 5’- GACACAGCAGTCACCAGAGGAT -3’ |
|  | Reverse 5’- TCACAACTGATGTCAAAGGAGCC -3’ |
| Human  MPO | Forward 5’- GAGCAGGACAAATACCGCACCA-3’ |
|  | Reverse 5’- AGAGAAGCCGTCCTCATACTCC -3’ |
| Human  MMP9 | Forward 5’- GCCACTACTGTGCCTTTGAGTC -3’ |
|  | Reverse 5’- CCCTCAGAGAATCGCCAGTACT -3’ |
| Human  METTL7A | Forward 5’- GTGCTCTGTGAAGAACCAGGAG -3’ |
|  | Reverse 5’- GATCCAGGACTTGTTGCCAGAAG -3’ |
| Human  METTL3 | Forward 5’- CTATCTCCTGGCACTCGCAAGA -3’ |
|  | Reverse 5’- GCTTGAACCGTGCAACCACATC -3’ |
| Human  METTL4 | Forward 5’- CTTGGTCTGTGGAGGTAGTTGC -3’ |
|  | Reverse 5’- CCAGTATAAGACCTTCGTAGGGC -3’ |
| Human  METTL5 | Forward 5’ - GCCCAAGCTACTTCTGGAACAG -3’ |
|  | Reverse 5’ - CCGATGCTAAGTACTCCACAACC -3’ |
| Human  METTL14 | Forward 5’- CTGAAAGTGCCGACAGCATTGG -3’ |
|  | Reverse 5’- CTCTCCTTCATCCAGATACTTACG -3’ |
| Human  WTAP | Forward 5’- GCAACAACAGCAGGAGTCTGCA -3’ |
|  | Reverse 5’- CTGCTGGACTTGCTTGAGGTAC -3’ |
| Human  FTO | Forward 5’- CCAGAACCTGAGGAGAGAATGG -3’ |
|  | Reverse 5’- CGATGTCTGTGAGGTCAAACGG -3’ |
| Human  YTHDF1 | Forward 5’- CAAGCACACAACCTCCATCTTCG -3’ |
|  | Reverse 5’- GTAAGAAACTGGTTCGCCCTCAT -3’ |
| Human  YTHDF2 | Forward 5’- TAGCCAGCTACAAGCACACCAC -3’ |
|  | Reverse 5’- CAACCGTTGCTGCAGTCTGTGT -3’ |
| Human  PLSCR1 | Forward 5’- ATATAATCAGCCAGTTGGAGCT -3’ |
|  | Reverse 5’- AAGGTAAAAGGTCTAGATGGCC -3’ |
| Human  CYBB | Forward 5’- CTCTGAACTTGGAGACAGGCAAA -3’ |
|  | Reverse 5’- CACAGCGTGATGACAACTCCAG -3’ |
| Human  NCF1 | Forward 5’- AATGGCAGGACCTGTCGGAGAA -3’ |
|  | Reverse 5’- CCTGTTCTCTGGATTGATCGCC -3’ |
| Human  β-actin | Forward 5’- GGCTACAGCTTCACCACCAC -3’ |
|  | Reverse 5’- TGCGCTCAGGAGGAGC -3’ |
| Human  GAPDH | Forward 5’- GTCTCCTCTGACTTCAACAGCG -3’ |
|  | Reverse 5’- ACCACCCTGTTGCTGTAGCCAA -3’ |
| Mouse  Il-17a | Forward 5’- CAGACTACCTCAACCGTTCCAC -3’ |
|  | Reverse 5’- TCCAGCTTTCCCTCCGCATTGA -3’ |
| Mouse  Tnf-α | Forward 5’- CACACTCACAAACCACCAAGTG -3’ |
|  | Reverse 5’- GCAGCCTTGTCCCTTGAAGA -3’ |
| Mouse  Il-1β | Forward 5’- TGCCACCTTTTGACAGTGATG -3’ |
|  | Reverse 5’- TGATGTGCTGCTGCGAGATT -3’ |
| Mouse  Il6 | Forward 5’- TACCACTTCACAAGTCGGAGGC -3’ |
|  | Reverse 5’- CTGCAAGTGCATCATCGTTGTTC -3’ |
| Mouse  Lcn2 | Forward 5’- ATGTCACCTCCATCCTGGTCAG -3’ |
|  | Reverse 5’- GCCACTTGCACATTGTAGCTCTG -3’ |
| Mouse  Cxcl1 | Forward 5’- TCCAGAGCTTGAAGGTGTTGCC -3’ |
|  | Reverse 5’- AACCAAGGGAGCTTCAGGGTCA -3’ |
| Mouse  Cxcl12 | Forward 5’- GGAGGATAGATGTGCTCTGGAAC -3’ |
|  | Reverse 5’- AGTGAGGATGGAGACCGTGGTG -3’ |
| Mouse  Camp | Forward 5’- CTTCAACCAGCAGTCCCTAGAC -3’ |
|  | Reverse 5’- GCCACATACAGTCTCCTTCACTC -3’ |
| Mouse  S100a8 | Forward 5’- CAAGGAAATCACCATGCCCTCTA -3’ |
|  | Reverse 5’- ACCATCGCAAGGAACTCCTCGA -3’ |
| Mouse  S100a9 | Forward 5’- TGGTGGAAGCACAGTTGGCAAC -3’ |
|  | Reverse 5’- CAGCATCATACACTCCTCAAAGC -3’ |
| Mouse  Mettl7a | Forward 5’- TGCTCGGTGAAGAACCAGGAGA -3’ |
|  | Reverse 5’- TCCAGGTAGACCGTTCATCTGC -3’ |
| Mouse  Gapdh | Forward 5’- CATCACTGCCACCCAGAAGACTG -3’ |
|  | Reverse 5’- ATGCCAGTGAGCTTCCCGTTCAG -3’ |

**Table S3. siRNA sequences list**

| **siRNA** | **sequences** |
| --- | --- |
| Human  METTL7A-1 | 5’- GCUGAGUGUUCGACUUGGAAUdTdT -3’ |
|  | 5’AUUCCAAGUCGAACACUCAGCdTdT -3’ |
| Human  METTL7A-2 | 5’- GUUGGUGCGCCCUCAUAUCUAdTdT -3’ |
|  | UAGAUAUGAGGGCGCACCAACdTdT -3’ |
| Human  METTL7A-3 | 5’- GGUUCACUGUGAUAUACAAdTdT -3’ |
|  | UUGUAUAUCACAGUGAACCdTdT -3’ |
| Human  PLSCR1-1 | 5’- GGAUUAGUGAAAGUCUCCUdTdT -3’ |
|  | AGGAGACUUUCACUAAUCCdTdT -3’ |
| Human  PLSCR1-2 | 5’- GACGCUGAUAACUUUGGAAdTdT -3’ |
|  | UUCCAAAGUUAUCAGCGUCdTdT -3’ |
| Human  PLSCR1-3 | 5’- CAGUAUCCACCGACAGCAUdTdT -3’ |
|  | AUGCUGUCGGUGGAUACUGdTdT -3’ |
| Human  YTHDF2-1 | 5’- CUGGAUAUAGUAGCAAUUAdTdT -3’ |
|  | 5’- UAAUUGCUACUAUAUCCAGdTdT -3’ |
| Human  YTHDF2-2 | 5’- CCAUUACUAGUAACAUCGUdTdT -3’ |
|  | 5’- ACGAUGUUACUAGUAAUGGdTdT -3’ |
| Human  YTHDF2-3 | 5’- GAUAUUCACCGUUCCAUUAdTdT -3’ |
|  | 5’- UAAUGGAACGGUGAAUAUCdTdT -3’ |

**Table S4. PLSCR1 m^6^A high-scoring prediction site primer sequence**

| **Primer** | **sequences** |
| --- | --- |
| PLSCR1 | Forward 5’- TGAACAGTGTGTGGTTGGCA -3’ |
|  | Reverse 5’- TGAGGAAACAGGCACCAATCA -3’ |
